# σ^P^-NagA-L1/L2 Regulatory Circuit Involved in *ΔompA_299-356_*-Mediated Increase in β-Lactam Susceptibility in Stenotrophomonas maltophilia

**DOI:** 10.1128/spectrum.02797-22

**Published:** 2022-11-09

**Authors:** Li-Hua Li, Cheng-Mu Wu, Chia-Lun Chang, Hsin-Hui Huang, Chao-Jung Wu, Tsuey-Ching Yang

**Affiliations:** a Department of Pathology and Laboratory Medicine, Taipei Veterans General Hospital, Taipei, Taiwan; b School of Medical Laboratory Science and Biotechnology, College of Medical Science and Technology, Taipei Medical University, Taipei, Taiwan; c Department of Biotechnology and Laboratory Science in Medicine, National Yang Ming Chiao Tung University, Taipei, Taiwan; d Department of Laboratory Medicine, National Taiwan University Hospital Hsin-Chu Branch, Hsinchu, Taiwan; University of Manitoba

**Keywords:** OmpA, sigma factor, beta-lactam resistance, peptiodglycan stress

## Abstract

OmpA, the most abundant porin in Stenotrophomonas maltophilia KJ, exists as a two-domain structure with an N-terminal domain of β-barrel structure embedded in the outer membrane and a C-terminal domain collocated in the periplasm. KJΔOmpA_299-356_, an *ompA* mutant of S. maltophilia KJ with a truncated OmpA devoid of 299 to 356 amino acids (aa), was able to stably embed in the outer membrane. KJΔOmpA_299-356_ was more susceptible to β-lactams than wild-type KJ. We aimed to elucidate the mechanism underlying the Δ*ompA*_299-356_-mediated increase in β-lactam susceptibility (abbreviated as “ΔOmpA_299-356_ phenotype”). KJΔOmpA_299-356_ displayed a lower ceftazidime (CAZ)-induced β-lactamase activity than KJ. Furthermore, KJ2, a L1/L2 β-lactamases-null mutant, and KJ2ΔOmpA_299-356_, a KJ2 mutant with truncated OmpA devoid of299 to 356 aa, had comparable β-lactam susceptibility. Both lines of evidence indicate that decreased β-lactamase activity contributes to the ΔOmpA_299-356_ phenotype. We analyzed the transcriptome results of KJ and KJΔOmpA_299-356_, focusing on PG homeostasis-associated genes. Among the 36 genes analyzed, the *nagA* gene was upregulated 4.65-fold in KJΔOmpA_299-356_. Deletion of the *nagA* gene from the chromosome of KJΔOmpA_299-356_ restored β-lactam susceptibility and CAZ-induced β-lactamase activity to wild-type levels, verifying that *nagA*-upregulation in KJΔOmpA_299-356_ contributes to the ΔOmpA_299-356_ phenotype. Furthermore, transcriptome analysis revealed that *rpoE* (Smlt3555) and *rpoP* (Smlt3514) were significantly upregulated in KJΔOmpA_299-356_. The deletion mutant construction, β-lactam susceptibility, and β-lactamase activity analysis demonstrated that σ^P^, but not σ^E^, was involved in the ΔOmpA_299-356_ phenotype. A real-time quantitative (qRT-PCR) assay confirmed that *nagA* is a member of the σ^P^ regulon. The involvement of the σ^P^-NagA-L1/L2 regulatory circuit in the ΔOmpA_299-356_ phenotype was manifested.

**IMPORTANCE** Porins of Gram-negative bacteria generally act as channels that allow the entry or extrusion of molecules. Moreover, the structural role of porins in stabilizing the outer membrane by interacting with peptidoglycan (PG) and the outer membrane has been proposed. The linkage between porin deficiency and antibiotic resistance increase has been reported widely, with a rationale for blocking antibiotic influx. In this study, a link between porin defects and β-lactam susceptibility increase was demonstrated. The underlying mechanism revealed that a novel σ^P^-NagA-L1/L2 regulatory circuit is triggered due to the loss of the OmpA-PG interaction. This study extends the understanding on the porin defect and antibiotic susceptibility. Porin defects may cause opposite impacts on antibiotic susceptibility, which is dependent on the involvement of the defect. Blocking the porin channel role can increase antibiotic resistance; in contrast, the loss of porin structure role may increase antibiotic susceptibility.

## INTRODUCTION

The outer membrane, a unique organelle of Gram-negative bacteria, protects them against harsh environments (1). Porins are the most abundant proteins in the outer membrane. They are designed for exchanging molecules across the outer membrane (2) and act as crucial factors in cell-to-cell signaling and environmental sensing. Porins are classified into the following two types according to their physiological roles: classical and slow porins (3). Classical porins serve as the entry point for molecules in a nonselective fashion (such as OmpF and OmpC of Escherichia coli and OmpK36 of Klebsiella pneumonia) (3, 4) or in a substrate-specific manner (such as LamB of E. coli and ScrY of Salmonella enterica subsp. Typhimurium) (5, 6). Relative to classical porins, molecular transportation is not the major function of slow porins because of their very low permeability. A classic example of a slow porin is the OmpA. OmpA is an abundant β-barrel porin highly conserved among bacterial species and is characterized as a two-domain structure with an N-terminal domain of β-barrel structure embedded in the outer membrane and a C-terminal OmpA-like globular domain located in the periplasmic space (7–10). Critical biological functions of the periplasmic globular domain of OmpA have been revealed, including noncovalent association with the peptidoglycan (PG) layer (11–13), thereby maintaining outer membrane integrity and signal transduction. OmpA is a multifaceted outer membrane protein (OMP) that is involved in a number of functions, such as adhesion, invasion, swimming, serum resistance, biofilm formation, and antibiotic resistance (14, 15), and serves as a receptor for pilus, bacteriophages, and bacteriocins (16). Furthermore, OmpA is an immune target that induces a host immune response; this feature makes OmpA the most popular vaccine candidate against Gram-negative pathogens (17).

Bacterial cell walls are essential for bacterial viability because they provide structural strength and counteract osmotic pressure in the cytoplasm. PG, which comprises sugars and amino acids, is a critical component of the cell wall (18). Given its uniqueness to bacteria, PG is a promising target for antibiotics, such as β-lactams. The target of β-lactam action is penicillin-binding proteins (PBPs), which participate in PG synthesis (19).

The known mechanisms responsible for β-lactam resistance include decreased outer membrane permeability, overexpression of efflux pumps, mutation of β-lactam targets, and overexpression of β-lactamase (20). β-Lactamase overexpression remains the primary mechanism used by Gram-negative bacteria to withstand β-lactam action. In some Gram-negative bacteria (such as Enterobacter cloacae, Citrobacter freundii, Pseudomonas aeruginosa, and Stenotrophomonas maltophilia), the inducible expression of chromosomally encoded β-lactamase is tightly linked to the cell wall recycling process (21). The cleaved PG sacculus in the periplasm is transported into the cytosol and further processed into different derivatives. Of these derivatives, 1,6-anhydro-MurNAc-peptides and UPD-MurNAc-pentapeptides function as activator and repressor ligands, respectively, which bind with AmpR and induce and repress the expression of β-lactamase genes, respectively (22–24).

Bacterial gene expression is often regulated at the transcriptional level. RNA polymerase (RNAP), an enzyme complex responsible for transcription, is essential to life. Bacterial RNAP consists of six subunits, as follows: α^I^, α^II^, β, β’, ω, and σ (25). The transcription process begins with the assembly of α^I^, α^II^, β, β’, and ω subunits into a core RNAP complex, and then a σ factor is recruited to form the holoenzyme. Bacteria generally harbor several different σ factors that specifically switch gene expression (26). The sigma factor recognizes cognate promoter sequences upstream of the genes that comprise the regulon of the σ factor (27). Based on their sequence, domain architecture, and function, σ factors fall into two distinct families, as follows: the σ^54^ factor (RpoN) family and σ^70^ family (RpoD) (27). The σ^70^ family factors have been classified into the following four groups: I, II, III, and IV. Group IV σ factors (or extracytoplasmic function [ECF] σ factors) are capable of sensing and responding to signals generated outside the cell or in the cell envelope (28, 29). ECF factors participate in several biological functions, such as envelope stress response, cell wall stress response, oxidative stress response, and iron transport (30).

Stenotrophomonas maltophilia, an opportunistic pathogen, is increasingly being recognized as an important cause of nosocomial infections. S. maltophilia is intrinsically resistant to several antibiotics because it possesses a number of antibiotic resistance determinants, such as β-lactamases, efflux pumps, and aminoglycoside-modifying enzymes (31). Thus, the challenge in the treatment of S. maltophilia infection is increasing.

S. maltophilia is intrinsically resistant to most β-lactams because of chromosomally encoded L1 and L2 β-lactamases. Of the β-lactams, ceftazidime (CAZ) and ticarcillin-clavulanic acid (TIM) are the choices used for treating S. maltophilia infection. L1 and L2 β-lactamase-inducible expression of S. maltophilia is linked to the disturbance of PG homeostasis (32), such as AmpC expression in E. cloacae, C. freundii, and P. aeruginosa (22). In addition to β-lactamase, non-β-lactamase-mediated β-lactam resistance in S. maltophilia is also reported. For example, the loss of function of *phoPQ* results in the alteration of outer membrane permeability, which is involved in the compromise of β-lactam resistance (32).

*OmpA* (Smlt0955) of S. maltophilia is known to be the highly expressed gene in logarithmically grown S. maltophilia (15, 33). The relationship between *ompA* deletion, swimming compromise, and conjugation failure has been reported in our recent study (15). In this study, we aimed to elucidate the relationship between OmpA defects and antibiotic susceptibility of S. maltophilia. Our findings revealed the involvement of *N*-acetylglucosamine-6-phosphate deacetylase (NagA) and a novel ECF σ factor (σ^P^) in the β-lactam susceptibility of S. maltophilia.

## RESULTS

### The truncated OmpA protein expressed in KJΔOmpA_299-356_ is embedded in the outer membrane.

**FIG 1:**
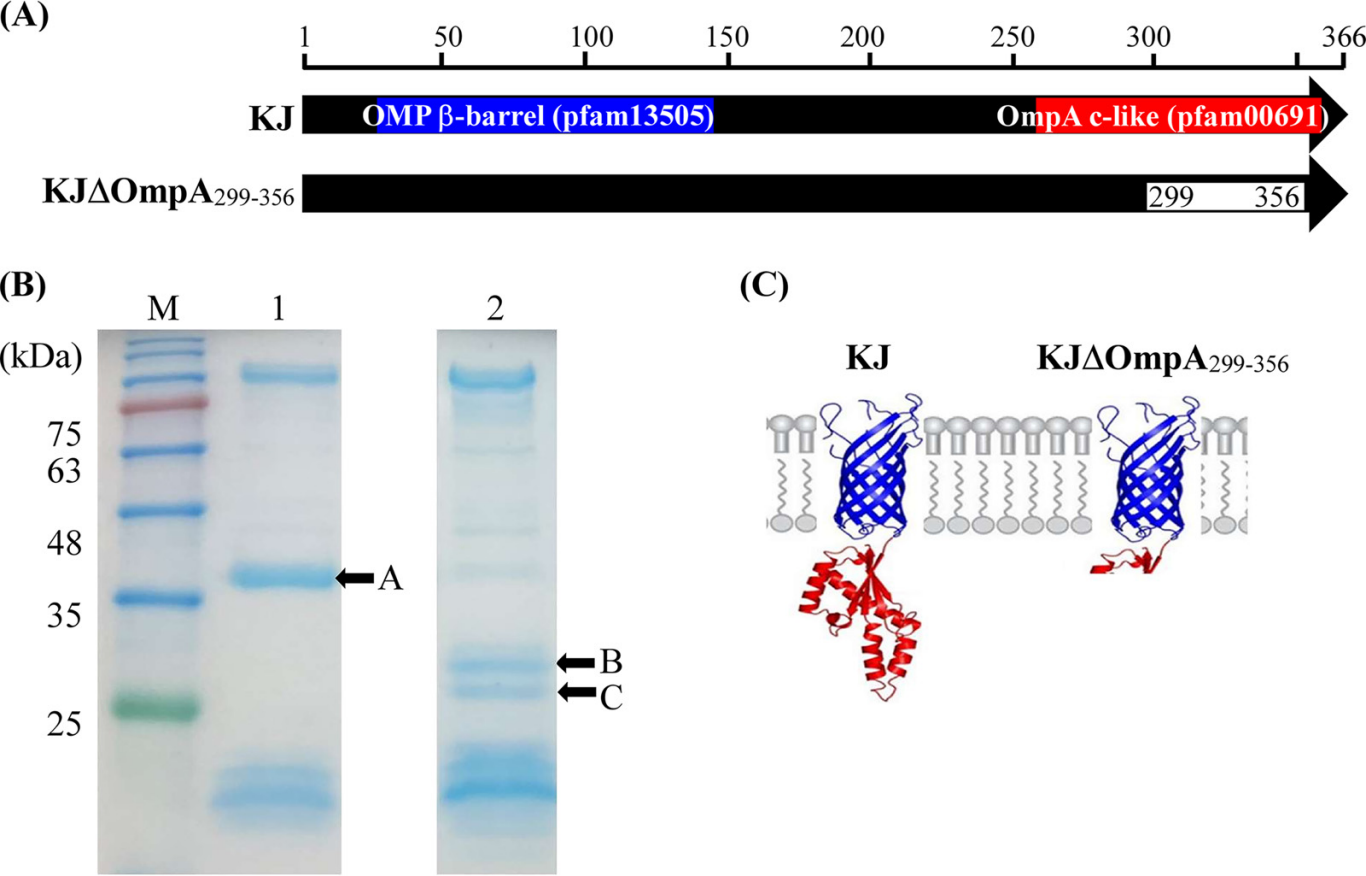
Construction strategy and outer membrane protein profiling of KJΔOmpA_299-356_, an *ompA* in-frame deletion mutant. (A) Diagram of conserved protein domains of OmpA and the deleted region of the *ompA* gene in KJΔOmpA_299-356_. The OMP β-barrel and OmpA c-like domains of the OmpA protein are marked in blue and red, respectively. The deleted region of the *ompA* gene is indicated as a white rectangle. (B) Outer membrane protein profiling of KJ and KJΔOmpA_299-356_. Outer membrane proteins were prepared from logarithmic-phase KJ and KJΔOmpA_299-356_ as described in the Materials and Methods and then separated by SDS-PAGE with a 5% stacking gel and a 15% separating gel. The gel was stained with 0.1% Coomassie brilliant blue and destained with 40% methanol-10% glacial acetic acid. Lane M, molecular weight standards; 1, KJ; 2, KJΔOmpA_299-356_. The black arrows indicate the protein excised for LC-MS/MS analysis. (C) An artistic impression of OmpA proteins in wild-type KJ and KJΔOmpA_299-356_ mutant.

KJΔOmpA, an in-frame *ompA* deletion mutant, was constructed in our previous study (15) in which the C-terminal OmpA c-like domain was partially deleted (299 to 356 amino acids) (Fig. 1A). For a more precise description of its characteristics, we renamed KJΔOmpA (15) as KJΔOmpA_299-356_ here. As the N-terminal β-barrel domain of OmpA remained intact in KJΔOmpA_299-356_, we wondered whether the truncated OmpA protein could be embedded in the outer membrane. The OMPs of KJ and KJΔOmpA_299-356_ were purified and subjected to sodium dodecyl sulfate-polyacrylamide gel electrophoresis (SDS-PAGE) analysis. A comparison between the OMP profiles of both strains revealed that KJΔOmpA_299-356_ was short of a protein band (band A in Fig. 1B) and had two additional protein bands compared to wild-type KJ (bands B and C in Fig. 1B). The three protein bands were excised from the gel and characterized using liquid chromatography-tandem mass spectrometry (LC-MS/MS). The masses and fragmentation patterns of band A correlated with OmpA, proving the correctness of KJΔOmpA_299-356_ (see Table S1 in the supplemental material). As determined using LC-MS/MS, band B was also identified as OmpA. It is worth mentioning that the fragmentation patterns of band B covered the N-terminal β-barrel domain of OmpA but not the predicted signal peptide (1- to 22-amino acid residues) or the deleted C-terminal region (see Table S2 in the supplemental material). The expected truncated OmpA protein of KJΔOmpA_299-356_ should have a molecular weight of 31.1 kDa, which matches the location of band B in the gel (Fig. 1Β). The LC-MS/MS results for band C revealed Smlt4119 as a candidate (see Table S3 in the supplemental material). Smlt4119 is predicted to be a 272-aa OMP with a 25-aa signal peptide. The predicted molecular weight of mature Smlt4119 (without the signal peptide) was 27.9 kDa, consistent with its position in the SDS-PAGE gel (Fig. 1B). Collectively, our results supported that the truncated OmpA protein of KJΔOmpA_299-356_ was able to stably embed in the outer membrane (Fig. 1C).

### KJΔOmpA_299-356_ is more susceptible to β-lactam than KJ.

Antibiotic susceptibilities of wild-type KJ and KJΔOmpA_299-356_ were examined. The antibiotics tested included β-lactam (ceftazidime [CAZ] and ticarcillin-clavulanic acid [TIM]), fluoroquinolone (ciprofloxacin, levofloxacin, and moxifloxacin), and trimethoprim-sulfamethoxazole, which are common choices for the treatment of S. maltophilia infection. Among the antibiotics tested, alteration in β-lactam susceptibility was the most apparent. Compared with wild-type KJ, KJΔOmpA_299-356_ reduced the MICs of TIM and CAZ (Table 1); however, the impact of truncated OmpA on susceptibility to quinolone and trimethoprim-sulfamethoxazole was mild and not significant (see Table S4 in the supplemental material).

**TABLE 1:** Antibiotic susceptibilities of S. maltophilia KJ and its derived mutants

Strain	MIC (μg/mL) of:^*a*^	
	CAZ	TIM
KJ	256	96
KJΔOmpA_299-356_	16	32
KJL2-OmpAΔOmpA_299-356_	>256	192
KJ2	0.094	0.094
KJ2ΔOmpA_299-356_	0.094	0.125
KJΔNagAΔOmpA_299-356_	>256	96
KJΔRpoEΔOmpA_299-356_	12	32
KJΔRpoPΔOmpA_299-356_	192	96
KJL2-RpoP	48	48
KJL2-RpoNΔOmpA_299-35_	24	32

a CAZ, ceftazidime; TIM, ticarcillin-clavulanate.

In our previous study, we demonstrated that KJΔOmpA_299-356_ is unable to obtain an exogenous plasmid by conjugation (15); thus, the plasmid-mediated complementation assay was inaccessible for KJΔOmpA_299-356_. Furthermore, from the transcriptome data (15), we noticed that the *L2* transcript level in KJΔOmpA_299-356_ had a 3.82-fold increase compared with that in the wild-type KJ. Therefore, we constructed an alternative *ompA* complementary strain KJL2-OmpAΔOmpA_299-356_, in which the complemented *ompA* gene was inserted downstream of the *L2* gene without disrupting the *L2* gene (see Fig. S1 in the supplemental material). The *ompA* gene insertion had no impact on *L2* expression, which was verified by qRT-PCR (data not shown). The susceptibilities of KJL2-OmpAΔOmpA_299-356_ to TIM and CAZ were examined, and reverted MIC values were observed (Table 1).

**FIG 2:**
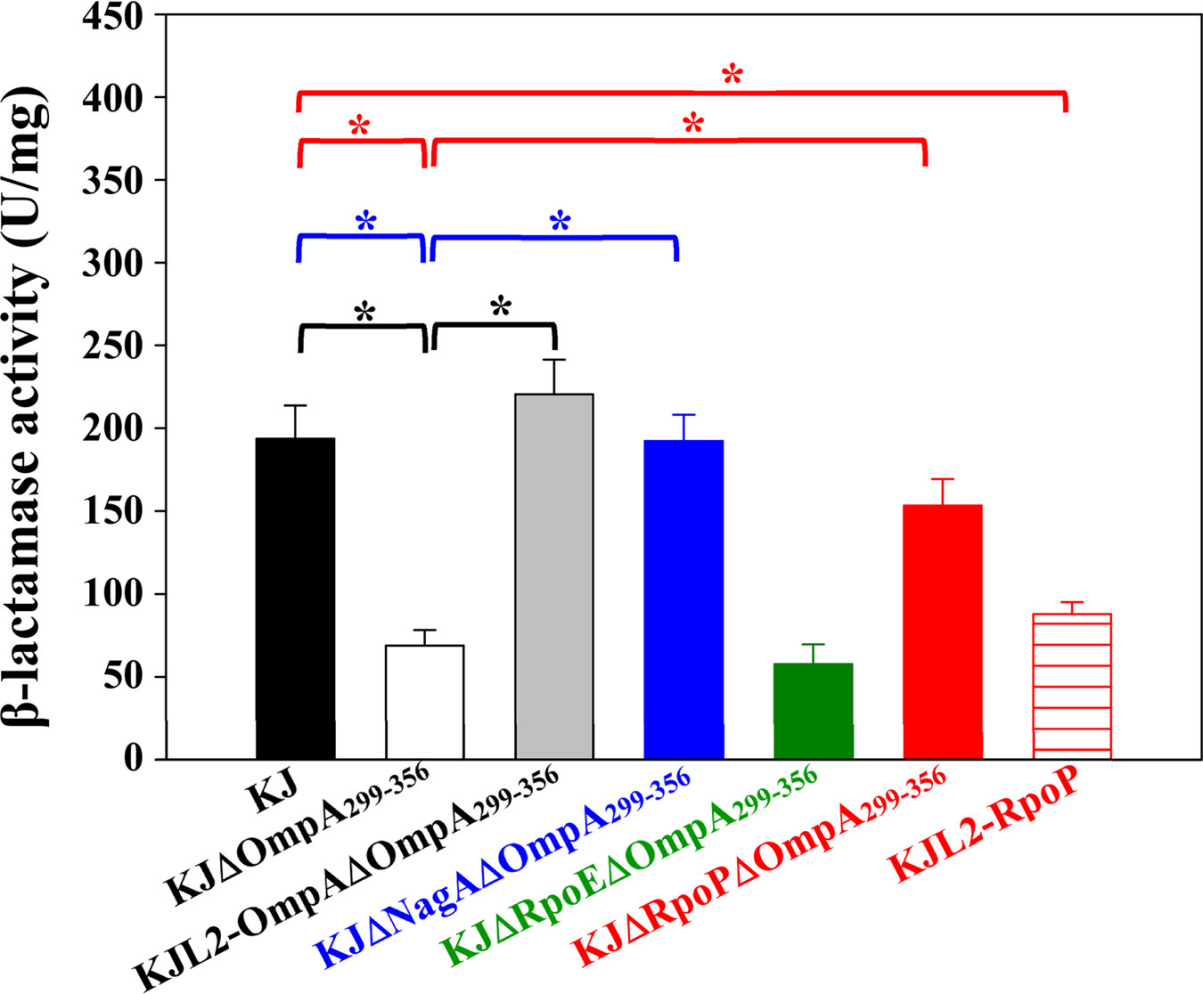
CAZ-induced β-lactamase activities of S. maltophilia KJ and its derived in-frame deletion constructs. Overnight-cultured bacterial cells were inoculated into fresh LB broth at an initial OD_450_ of 0.15 and subcultured for 3 h. Induction was carried out by adding CAZ of 1/4 MIC for 1 h, and the induced β-lactamase activities were determined. Bars represent the average values from three independent experiments. Error bars represent the SEM. *, *P < *0.001; significance was calculated by Student’s *t* test. CAZ, ceftazidime.

The known mechanisms responsible for the β-lactam resistance of S. maltophilia can be classified into β-lactamase-mediated resistance and non-β-lactamase-mediated resistance (33, 34). The β-lactamase activities of KJ, KJΔOmpA_299-356_, and KJL2-OmpAΔOmpA_299-356_ were determined to assess the involvement of β-lactamases in the Δ*ompA*_299-356_-mediated increase in β-lactam susceptibility. Compared with wild-type KJ, KJΔOmpA_299-356_ had lower CAZ-induced β-lactamase activity and KJL2-OmpAΔOmpA_299-356_ exhibited comparable activities (Fig. 2). Next, non-β-lactamase-mediated resistance was further assessed in the L1 and L2 double deletion mutant, KJ2 (35). The *ΔompA_299-356_* allele was introduced into the chromosome of KJ2 to yield KJ2ΔOmpA_299-356_. KJ2ΔOmpA_299-356_ and the parental strain KJ2 displayed comparable susceptibility to TIM and CAZ (Table 1). Collectively, decreased β-lactamase activity was the dominant factor responsible for the Δ*ompA*_299-356_-mediated increase in β-lactam susceptibility.

### *NagA* (Smlt4020) upregulation in KJΔOmpA_299-356_ is involved in the Δ*ompA*_299-356_-mediated increase in β-lactam susceptibility.

**FIG 3:**
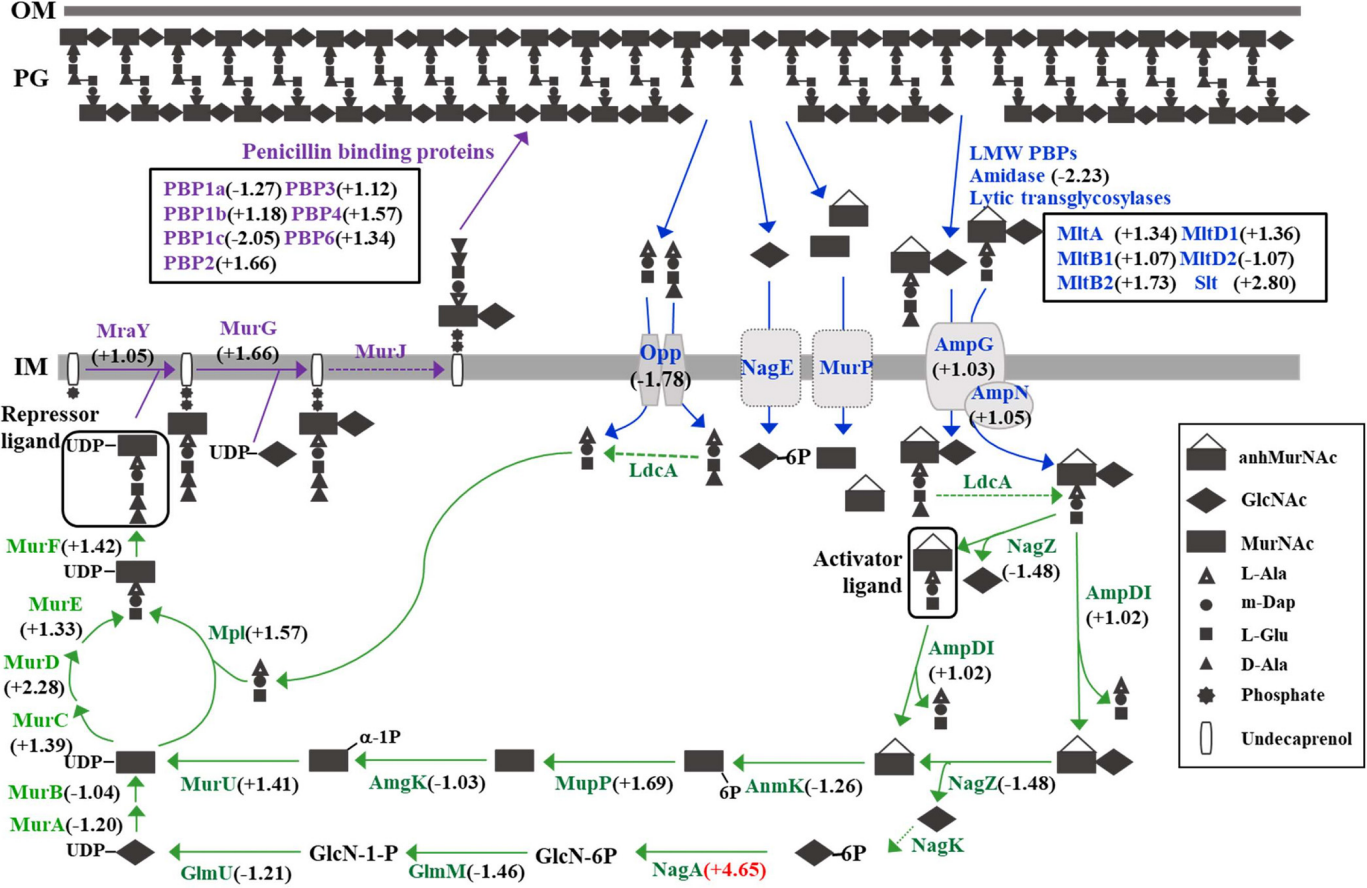
Schematic representation of PG biosynthesis, catabolism, and recycling, and the comparative transcriptome analysis in S. maltophilia KJ and its derived *ompA* mutant, KJΔOmpA_299-356_. The PG homeostasis model of S. maltophilia is proposed based on the known model from P. aeruginosa. The PG biosynthesis (labeled in purple) and PG catabolism (labeled in blue) majorly occur in the periplasm. AmpNG permease system transports the PG degradation fragments from periplasm into cytosol for further PG recycling (labeled in green). The dashed lines indicate the homologs of P. aeruginosa are not identified from S. maltophilia K279a genome via BLAST analysis. The RNA-Seq transcriptome analysis of KJ and KJΔOmpA_299-356_ was performed. The number in each bracket indicates the fold of gene expression change in wild-type KJ and KJΔOmpA_299-356_. Positive values indicate the gene expression in KJΔOmpA_299-356_ is higher than that in wild-type KJ, and negative values represent the gene expression in KJ is higher than that in KJΔOmpA_299-356_.

**FIG 4:**
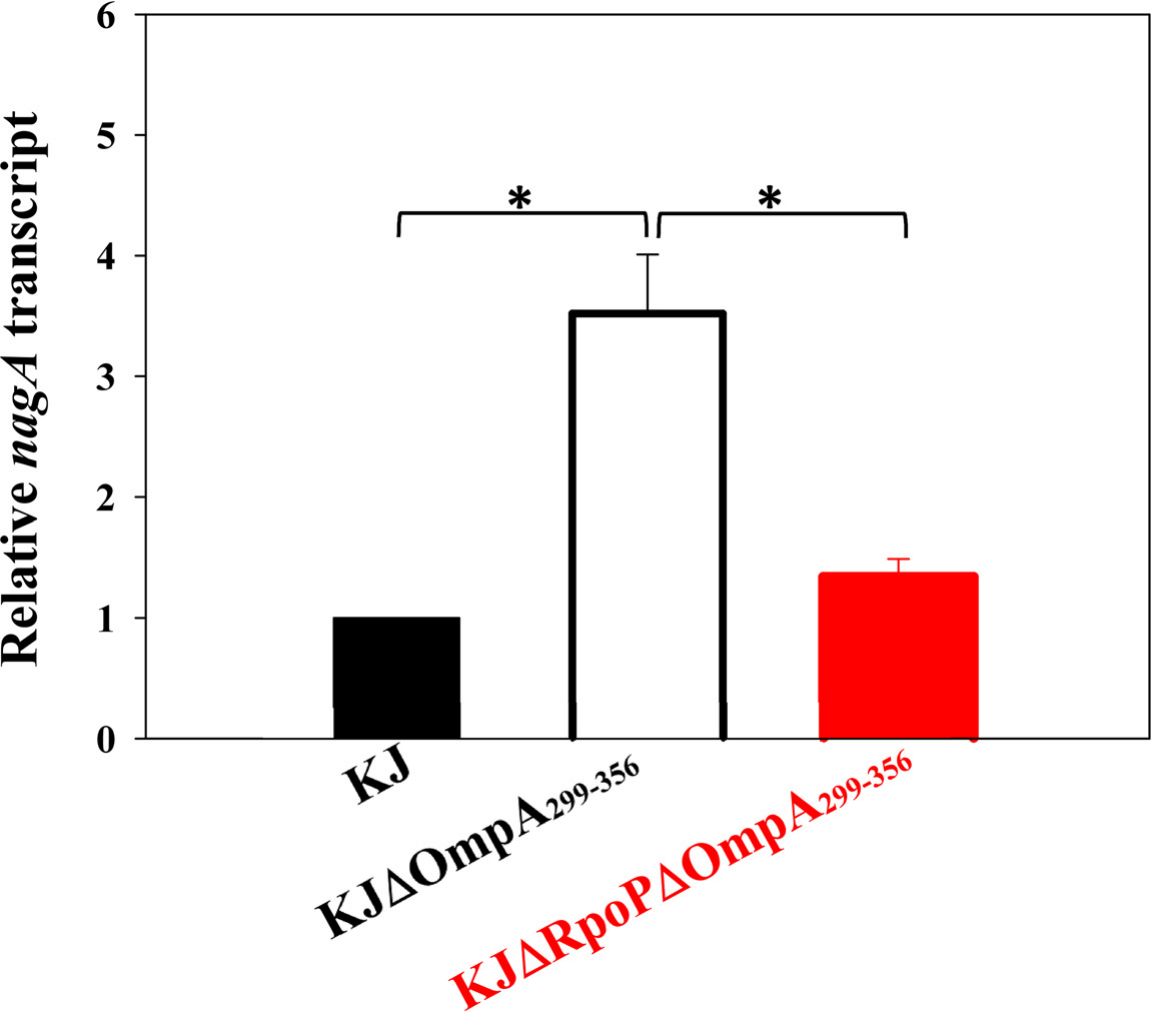
The *nagA* transcript level of wild-type KJ and its derived mutants. Overnight culture of strains tested were inoculated into fresh LB at an initial OD_450_ of 0.15 and aerobically grown for 5 h. The *nagA* transcript level was determined via qRT-PCR using NagAQ103-F/R primers. Relative transcript level was normalized to the transcript level of KJ cells. Bars represent the average values from three independent experiments. Error bars represent the standard error of the mean. *, *P < *0.05; significance was calculated by Student’s *t* test.

The RNA sequencing (RNA-Seq) transcriptome assays of KJ and KJΔOmpA_299-356_ were conducted and validated in our previous study (15) (see Table S5 in the supplemental material). To elucidate the mechanism underlying the Δ*ompA*_299-356_-mediated increase in β-lactam susceptibility, we reanalyzed the transcriptome data (15) (Table S5). It is commonly recognized that PG homeostasis is linked to the expression of chromosomally encoded β-lactamase genes in S. maltophilia (32); thus, genes associated with PG homeostasis were surveyed. The PG homeostasis model for S. maltophilia has been proposed in our previous study, and there are at least 39 genes involved (32). Based on this model, we re-examined the transcript levels of the 39 genes from previous transcriptome data (15) (Table S5). We defined significance as the absolute fold change in TPM equal to or greater than 3. Among the 36 genes examined, *nagA* (Smlt4020) was significantly upregulated (Fig. 3; see Table S6 in the supplemental material). The expression of *nagA* in KJ and KJΔOmpA_299-356_ was validated using qRT-PCR (Fig. 4). The protein encoded by Smlt4020 is annotated as *N*-acetylglucosamine-6-phosphate deacetylase (NagA), which catalyzes the deacetylation of *N*-acetylglucosamine-6-phosphate (GlcNAc-6P) to glucosamine-6-phosphate (GlcN-6P). GlcN-6P is then used in two main pathways in bacteria, as follows: the PG recycling pathway and glycolysis pathway (36).

To clarify whether *nagA* upregulation in KJΔOmpA_299-356_ was responsible for the Δ*ompA*_299-356_-mediated increase in β-lactam susceptibility, a *nagA* deletion mutation was introduced into KJΔOmpA_299-356_. Introduction of the *ΔnagA* allele into KJΔOmpA_299-356_ restored β-lactam resistance (Table 1) and β-lactamase activity (Fig. 2), supporting that *nagA* overexpression in KJΔOmpA_299-356_ contributes to Δ*ompA*_299-356_-mediated increase in β-lactam susceptibility.

### *RpoP* (*Smlt3514*) upregulation in KJΔOmpA_299-356_ is involved in the Δ*ompA*_299-356_-mediated increase in β-lactam susceptibility.

**FIG 5:**
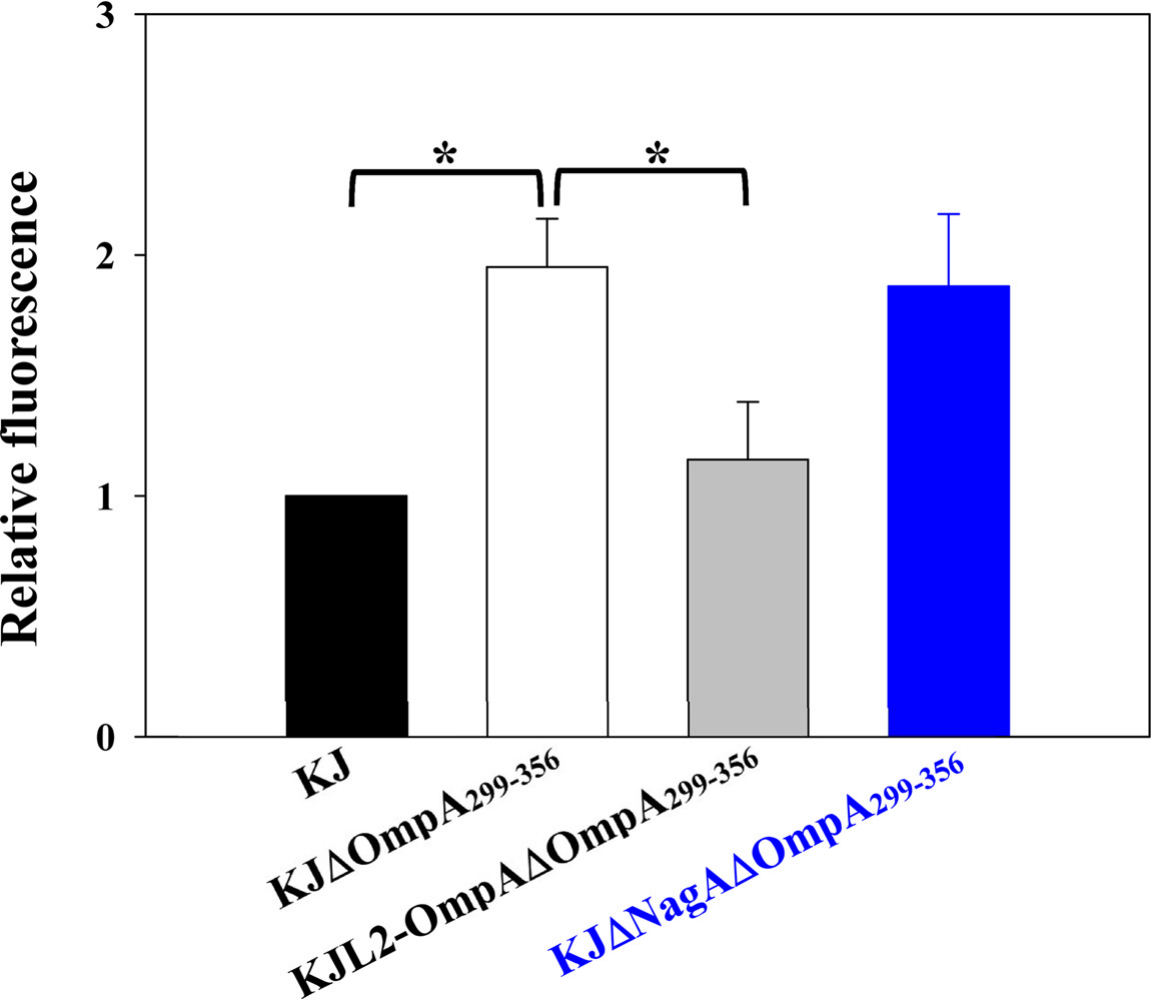
1-*N*-Phenylnaphthylamine (NPN) uptake assay. The 100-μL bacterial cells tested were pipetted into 96-well microtiter plates, and NPN was added to a final concentration of 15 μM. Fluorescence was monitored by fluorescence spectrophotometer at excitation and emission wavelengths of 355 nm and 402 nm, respectively. Relative fluorescence level was normalized to KJ cells. Bars represent the average values from three independent experiments. Error bars represent the standard error of the mean. *, *P < *0.05; significance was calculated by Student’s *t* test.

Given that OmpA is the most abundant OMP in wild-type KJ, we speculated that *ompA* deletion might affect outer membrane integrity. Thus, the outer membrane destabilization of KJΔOmpA_299-356_ was investigated using the 1-N-phenylnaphtylamine (NPN) uptake assay. NPN is an uncharged lipophilic dye with weak fluorescence in aqueous environments and great fluorescence in hydrophobic environments, such as the cell membrane. If the outer membrane integrity of KJΔOmpA_299-356_ is compromised, NPN dye is integrated into the inner membrane. The NPN assay demonstrated that the level of fluorescence intensity of KJΔOmpA_299-356_ was 1.95-fold higher than that of wild-type KJ and nearly reverted to the wild-type level in the complementary strain KJL2-OmpAΔOmpA_299-356_ (Fig. 5). It was worth mentioning that the fluorescence detected from KJΔNagAΔOmpA_299-356_ was comparable to that from KJΔOmpA_299-356_ (Fig. 5), indicating that *nagA* deletion from KJΔOmpA_299-356_ hardly reverted the outer membrane defect.

Extracytoplasmic function (ECF) σ factors provide a means of regulating gene expression in response to extracytoplasmic stress, such as imbalance of the outer membrane, peptidoglycan, and inner membrane (37). Given that the membrane integrity was compromised in KJΔOmpA_299-356_, the involvement of the ECF σ factor was considered. There were 16 annotated ECF σ factors in the S. maltophilia K279a genome (38). Transcriptome profiles of the 16 ECF σ factors in wild-type KJ and KJΔOmpA_299-356_ (15) (Table S5) were investigated. Among the 16 ECF σ factors assayed, two sigma factors, namely, *Smlt3514* and *rpoE*, displayed significant expression differences with 3.15- and 5.58-fold upregulation in KJΔOmpA_299-356_, respectively (Table 2). Based on the following results, we designated *Smlt3514* as *rpoP* (P meaning PG) here.

**TABLE 2:** Transcriptome analysis of ECF σ factor genes in wild-type KJ and *ompA* mutant, KJΔOmpA_299-356_

Locus	Protein	TPM^*a*^ of		Fold change^*b*^
		KJ	KJΔOmpA_299-356_	
Smlt0855	ECF σ factor	14.17	19.88	+1.40
Smlt1269	ECF σ factor	9.79	12.56	+1.28
Smlt1349	FecI-like σ factor	85.82	124.59	+1.45
Smlt1750	FecI-like σ factor	4.81	8.64	+1.80
Smlt2004	ECF σ factor	79.35	57.35	−1.38
Smlt2377	ECF σ factor	63.83	23.17	−2.75
Smlt2513	ECF σ factor	1.44	1.08	−1.34
Smlt2664	FecI-like σ factor	8.25	3.21	−2.57
Smlt2716	FecI-like σ factor	17.87	14.17	−1.26
Smlt2848	FecI-like σ factor	0	0	ND
Smlt2935	ECF σ factor	0.99	0	ND
Smlt3223	ECF σ factor	2.47	2.23	−1.11
Smlt3514	σP	49.65	156.39	+3.15
Smlt3555	σE	434.09	2,424.29	+5.58
Smlt3900	ECF σ factor	11.04	11.05	1.00
Smlt4579	ECF σ factor	0	0	ND

a TPM, transcripts per kilobase million.

b Negative fold changes represent genes that were significantly downregulated in KJΔOmpA_299-356_, whereas positive fold changes represent upregulation in KJΔOmpA_299-356_. ND, not determined.

The Smlt3514 gene encodes a 192-aa sigma-70 family, ECF subfamily RNA polymerase sigma factor. A survey on the 14 sequenced S. maltophilia strains revealed that this gene is completely conserved in this species, with an intraspecies protein identity of 93 to 100%. To clarify the relationship between σ^P^, σ^E^, and Δ*ompA*_299-356_-mediated increase in β-lactam susceptibility, we constructed *rpoE* and *rpoP* in-frame deletion mutants of KJΔOmpA_299-356_ (KJΔRpoEΔOmpA_299-356_ and KJΔRpoPΔOmpA_299-356_) and examined the β-lactam susceptibility and β-lactamase activity of these mutants. Compared with KJΔOmpA_299-356_, KJΔRpoPΔOmpA_299-356_, but not KJΔRpoEΔOmpA_299-356_, almost restored β-lactam resistance and β-lactamase activity to wild-type levels (Table 1, Fig. 2).

### *NagA* upregulation in KJΔOmpA_299-356_ is σ*^P^* dependent.

The next question we wondered was whether *nagA* is a member of the σ^P^ regulon. Thus, the *nagA* transcript level in KJΔRpoPΔOmpA_299-356_ was determined by qRT-PCR. Compared with that in wild-type KJ, the *nagA* transcript level of KJΔOmpA_299-356_ showed a 3.52- ± 0.49-fold increase and reverted to the wild-type level when *ΔrpoP* allele was introduced into the chromosome of KJΔOmpA_299-356_ (Fig. 4).

Based on the above results, we conclude that the Δ*ompA*_299-356_-mediated increase in β-lactam susceptibility seems to be attributed to the σ^P^-NagA-L1/L2 regulatory circuit. To further confirm this possibility, we constructed an *rpoP-*overexpression strain, KJL2-RpoP, in which the *rpoP* gene was inserted downstream of the *L2* gene (Fig. S1). Compared with wild-type KJ, KJL2-RpoP was more susceptible to β-lactams (Table 1) and had a decreased CAZ-induced β-lactamase activity (Fig. 2).

## DISCUSSION

Outer membrane porins are important channels for the influx of nutrients, hydrophilic molecules, and some antibiotics. The linkage between porin deficiency and β-lactam resistance has been reported widely; some examples are OmpK35 and OmpK36 of Klebsiella pneumoniae (39), OmpF of Serratia marcescens (40), and OprD and OprF of P. aeruginosa (41, 42). These porins (OmpK, OmpF, OprD, or OprF) are classical ones that generally function as outlets for molecule entrance or extrusion (43), and inactivation of these porins generally results in an increase in antibiotic resistance. Nevertheless, the OmpA is a slow porin. Smani et al. (44) found that *ompA* inactivation of A. baumannii decreased the β-lactam MICs and proposed that OmpA participates in antimicrobial extrusion, but they did not verify this assumption. The rationale proposed by Smani et al. (44) was based on the concept of OmpA as a transport channel for antibiotics. In this study, we revealed that the loss of function of OmpA of S. maltophilia compromised β-lactam resistance and disclosed the involvement of the σ^p^-NagA-L1/L2 regulatory circuit in this phenotype. Our findings emphasize the role of OmpA in stabilizing the PG layer, which is not limited to molecule transport. Disruption of PG homeostasis causes extracytoplasmic stress, which leads to ECF sigma factor activation and increases β-lactam susceptibility.

OmpA adopts a two-domain structure, with an N-terminal β-barrel embedded in the outer membrane (8) and a C-terminal globular domain in the periplasm (9, 10). Critical biological functions of the periplasmic C-terminal domain of OmpA have been revealed, including noncovalent association with the PG scaffold (12) and in complex with the RcsF lipoprotein (13). The stable binding of PG to the C-terminal domain of OmpA is a recognition mechanism for Gram-negative bacteria to maintain cell wall integrity (12). Structural analysis of Acinetobacter baumannii OmpA clearly indicated that Asp271 and Arg286 are key residues for OmpA and PG interactions (10). By protein alignment between OmpAs of A. baumannii and S. maltophilia, we found that the two residues are well conserved in S. maltophilia OmpA (Asp294 and Arg309) (see Fig. S2 in the supplemental material) and that Arg309 was deleted in KJΔOmpA_299-356_. Thus, it is not surprising that a loss of the C-terminal domain of OmpA may cause cell wall and/or cell envelope stress, which then triggers the activation of an ECF σ factor. As revealed in this study, KJΔOmpA_299-356_ exerted an effect on the upregulation of *rpoP*. The stimulus that triggers the upregulation of *rpoP* can be a compromise of PG stability caused by the loss of interaction between PG and the OmpA C-terminal domain, which can be regarded as a type of PG stress. This is the reason why we designate Smlt3514 as *rpoP*. Since the *ompA* mutant constructed in this study is a partial OmpA deletion mutant with an intact β-barrel component, we cannot immediately rule out the possibility that the β-barrel domain of OmpA is also required for the activation of σ^P^, in addition to the loss of the C-terminal domain.

The SDS-PAGE OMP profiling revealed that an OMP encoded by Smlt4119 was invisible in wild-type KJ but obviously increased in KJΔOmpA_299-356_ (Fig. 1B). The Smlt4119 protein is annotated as a hypothetical protein in the sequenced S. maltophilia genome and is not homologous to other known OMPs. The question of whether the increased Smlt4119 protein level has an impact on the *ΔompA_299-356_*-mediated increase in β-lactam susceptibility is not immediately clear right now. However, KJΔNagAΔOmpA_299-356_ and KJΔRpoPΔOmpA_299-356_ displayed comparable β-lactam susceptibility to wild-type KJ (Table 1), highly supporting that σ^P^-NagA-L1/L2 is the major regulatory circuit involved in the *ΔompA*_299-356_-mediated increase in β-lactam susceptibility.

Recently, we have demonstrated that *rpoN* was downregulated in KJΔOmpA_299-356_, which results in the swimming compromise of KJΔOmpA_299-356_ (15). In this article, we further elucidated that *rpoP* upregulation in KJΔOmpA_299-356_ contributes to a Δ*omp*_299-356_-mediated increase of β-lactam susceptibility. We were curious whether an interconnection between σ^N^- and σ^P^-mediated regulations happens. First, the β-lactam susceptibility of KJL2-RpoNΔOmpA_299-356_, a *rpoN* complementation strain of ΔOmpA_299-356_, was investigated. KJL2-RpoNΔOmpA_299-356_ exhibited comparable β-lactam susceptibility to KJΔOmpA_299-356_ (Table 1), indicating that *rpoN* downregulation in KJΔOmpA_299-356_ is less related to the Δ*ompA*_299-356_-mediated increase of β-lactam susceptibility. In addition, KJΔRpoPΔOmpA_299-356_ and KJΔOmpA_299-356_ displayed comparable swimming motility (see Fig. S3 in the supplemental material), which is less support for the involvement of σ^P^ in Δ*ompA*_299-356_-mediated swimming compromise. Collectively, σ^N^- and σ^P^-mediated regulations in KJΔOmpA_299-356_ appeared to independently link to the phenotypes of swimming compromise and β-lactam susceptibility increase.

PG recycling is a process whereby degraded PGs are recovered and made available for bacterial cells to synthesize more PG or to use it as an energy source. NagA is an enzyme involved in PG recycling (Fig. 3). NagA catalyzes the conversion of GlcNAc-6P to GlcN-6P, and GlcN-6P is used in PG recycling or glycolysis pathways. In the PG recycling pathway, GlcN-6P is subsequently processed into UPD-MurNAc-pentapeptide, which acts as a repressor ligand to bind with AmpR. UPD-MurNAc-pentapeptide-bound AmpR functions as a repressor of chromosomal β-lactamase genes expression (22). Based on this rationale, we propose an explanation for the role of *nagA* in the *ΔompA*-mediated increase in β-lactam susceptibility. The *nagA* upregulation in KJΔOmpA_299-356_ may increase the intracellular levels of UPD-MurNAc-pentapeptides, which attenuates the association between 1,6-anhydro-MurNAc-peptides and AmpR. This finding may explain why the β-lactamase activity of KJΔOmpA_299-356_ decreased.

The linkage between σ factor and β-lactam resistance has been reported in several bacteria, for example σ^P^ in Bacillus anthracis, Bacillus cereus, and Bacillus thuringiensis (45, 46); σ^M^, σ^X^, and σ^A^ in Bacillus subtilis (47, 48); σ^B^ in Staphylococcus aureus (49); *algT/U* in Pseudomonas aeruginosa (50); ECF-10 in Pseudomonas putida (51); and σ^E7^ and σ^H3^ in Azospirillum baldaniorum (52). The σ^P^ of S. maltophilia displayed 25 to 27% and 46 to 54% protein identity and similarity, respectively, to the σ^P^ of *Bacillus* spp. Some sigma factors, such as σ^P^ and σ^M^, are modulated by anti-σ^P^ via protein-protein interactions (45, 46). These σ factors are generally held inactive by anti-σ factors, and the genes encoding σ factor and anti-σ factor are organized into an operon (53, 54). We surveyed the genomic organization surrounding *rpoP*, but none of the open reading frames (ORFs) exhibited any homology to the anti-σ factor. However, interestingly, we noticed that the genes (Smlt3513 and Smlt3512) downstream of *rpoP* encode a 177-aa and a 293-aa hypothetical protein, respectively. The *rpoP*, Smlt3513, and Smlt3512 genes have the same orientation. *RpoP* and *Smlt3513* genes have a 4-bp overlapping, and *Smlt3513* and *Smlt3512* genes are separated by a 10-bp intergenic region. Based on this observation, we speculate that *rpoP-Smlt3513-Smlt3512* may form an operon and Smlt3513 and Smlt3512 may participate in the PG stress signal transduction from the periplasm into cytoplasm and then activate the cytoplasmic σ^P^.

**FIG 6:**
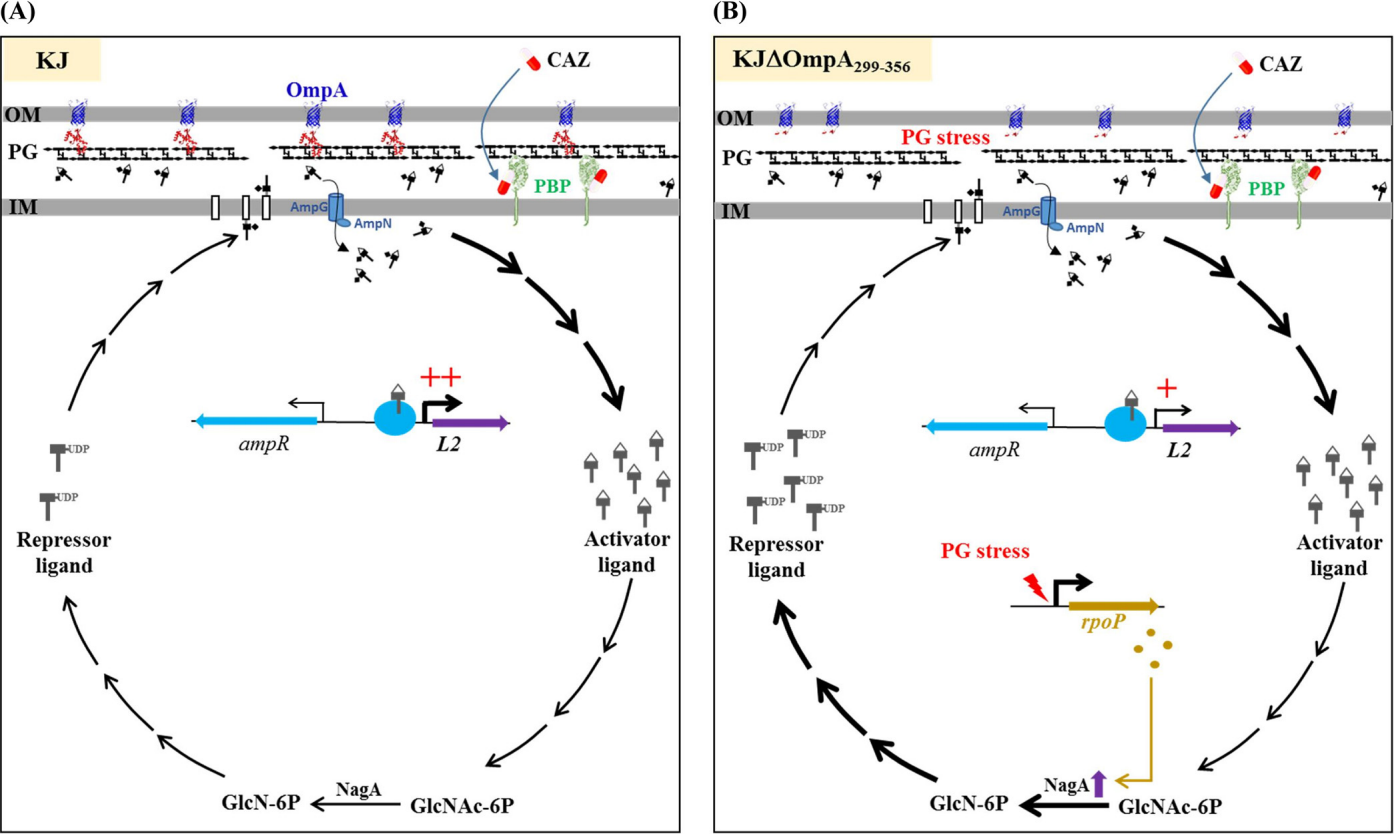
Model of the *rpoP-nagA-L1/L2* regulatory circuit in the *ΔompA*_299-356_-mediated increment of β-lactam susceptibility of Stenotrophomonas maltophilia. (A) The PG scaffold of Gram-negative bacteria is anchored noncovalently to the outer membrane via the C-terminal domain of OmpA proteins. The OmpA-PG interaction controls the stability of the cell wall. Ceftazidime (CAZ), a β-lactam antibiotic, targets on the penicillin binding protein (PBP) and blocks PG cross-linking. Accumulated murein sacculus is transported into the cytosol via the AmpNG permease system and further processed into 1,6-anhydro-MurNAc-pentapeptides (activator ligands). The activator ligands compete with UDP-MurNAc-pentapeptides (repressor ligands) for a binding site on AmpR. As AmpR is bound with activator ligands, it functions as a transcriptional activator, inducing the expression of L1 and L2 β-lactamases (L2 as a representative). (B) In the KJΔOmpA_229-356_ mutant, the C terminus-deleted OmpA loses the interaction with PG layers, generating a PG stress. PG stress triggers the upregulation of σ^P^, which then increases the expression of *nagA*. The upregulated NagA activity may favor the formation of repressor ligands. The increased repressor ligands are though to partially displace activator ligands from AmpR and attenuate CAZ-induced β-lactamases expression.

The genetic indications presented here lead to a model in which a σ^P^-NagA-L1/L2 regulatory circuit is responsible for the increase in β-lactam susceptibility in KJΔOmpA_299-356_. OmpA has a two-domain structure consisting of a β-stranded N-terminal domain and a globular C-terminal domain (16). The stable interaction between the C-terminal domain of OmpA and PG is critical for the maintenance of cell wall integrity (12) (Fig. 6A). When S. maltophilia KJ is challenged with CAZ, PG homeostasis is disturbed and abundant 1,6-anhydro-MurNAc-pentapeptides (activator ligands) dominate the binding with AmpR, which leads to L1/L2 β-lactamases upregulation and enhances β-lactam resistance (Fig. 6A). In the KJΔOmpA_229-356_ mutant, the OmpA protein lacks 229- to 356-amino acid residues but retains an intact N-terminal β-barrel domain, which allows it to assemble into the outer membrane. Due to the deletion of the C-terminal domain, OmpA loses its interaction with PG layers, which destroys PG stability and generates PG stress. In response to PG stress, ECF sigma factor σ^P^ is upregulated, which increases the expression of NagA. High expression of NagA may favor the formation of UPD-MurNAc-pentapeptides (repressor ligands). In such an instance, increased repressor ligands would alter the repressor ligand/activator ligand ratio, decrease the association of activator ligands and AmpR, and result in the reduction of β-lactam-induced β-lactamase activity (Fig. 6B).

## MATERIALS AND METHODS

### Bacterial strains, plasmids, and primers.

The bacterial strains, plasmids, and primers used in this study were summarized in Table S7 in the supplemental material.

### OMP preparation and SDS-PAGE.

The outer membrane proteins of the mid-log-phase bacterial cells were prepared as described previously (55). The OMPs were separated by sodium dodecyl sulfate-polyacrylamide gel electrophoresis (SDS-PAGE) in a 15% polyacrylamide gel. The gel was stained using 0.1% Coomassie brilliant blue R250 (Bio-Rad) and destained with 40% methanol-10% glacial (acetic acid) for the visualization of proteins.

### Construction of deletion mutants.

The deletion mutants were constructed using double crossover homologous recombination as described previously (56). Two DNA fragments flanking the *rpoE* were PCR amplified using primer sets RpoEN-F/RpoEN-R and RpoEC-F/RpoEC-R (Table S7) and then subsequently cloned into pEX18Tc to generate pΔRpoE (Table S7). The intact *nagA* and *rpoP* genes were amplified by PCR using primer sets NagA-F/NagA-R and RpoP-F/RpoP-R (Table S7) and then cloned into pEX18Tc, yielding pEXNagA and pEXRpoP, respectively. The pΔNagA and pΔRpoP (Table S7) were generated by removing a 432-bp PstI-PstI and a 369-SalI-SlaI DNA fragments from plasmid pEXNagA and pEXRpoP, respectively. Plasmids pΔRpoE, pΔNagA, and pΔRpoP were transferred into S. maltophilia KJ by conjugation. The plasmid’s conjugation, transconjugant’s selection, and mutant’s confirmation were carried out as described previously (56).

### Construction of KJL2-OmpAΔOmpA_299-356_ and KJL2-RpoP.

As the conjugation for the plasmid transportation is inapplicable in KJΔOmpA_299-356_ (15), an alternative strategy was designed for gene expression in KJΔOmpA_299-356_ by chromosomally inserting the *ompA* and *rpoP* genes, respectively, downstream *L2* gene without disrupting any gene. The *L2* and the inserted gene form an operon-like configuration, and the inserted gene is expressed inducibly upon β-lactam challenge (Fig. S1). The 503- and 547-bp DNA fragments containing the C terminus of the *L2* gene and downstream of the *L2* gene were obtained by PCR using the primer sets of HH1N-F/HH1N-R and HH1C-F/HH1C-R (Table S7), respectively, and then subsequently cloned into pEX18Tc, yielding plasmid pEXHH1 (Table S7). The multiple cloning sites (SphI/PstI/SalI/XbaI/BamHI/SmaI/KpnI/SacI) of pEX18Tc were conserved in plasmid pEXHH1 for cloning the exotic gene intended to be expressed. The intact *ompA* and *rpoP* genes were amplified by PCR using the primers sets HHOmpA-F/HHOmpA-R and HHRpoP-F/HHRpoP-R (Table S7) and then cloned into pEXHH to generate pEXHH1-OmpA and pEXHH1-RpoP (Table S7), respectively. The *ompA* and *rpoP* genes in pEXHH1-OmpA and pEXHH1-RpoP were inserted onto the intergenic region (IG) downstream the L2 gene of S. maltophilia KJ via double crossover homologous recombination as described previously (56) to yield KJL2-OmpA and KJL2-RpoP, respectively. The prototype chromosomal *ompA* gene was deleted from KJL2-OmpA by double crossover homologous recombination, and KJL2-OmpAΔOmpA_299-356_ was obtained.

### Antibiotic susceptibility test.

The bacterial susceptibilities to antibiotics were determined by the Etest strips (bioMérieux, Marcy I’Etoile, France), according to the manufacturer’s instructions.

### β-Lactamase activity determination.

The β-lactamase activity was determined using the chromogenic substrate nitrocefin (Δε = 20,500 M^−1^ · cm^−1^ at 486 nm) as the substrate as described previously (56). One unit of enzyme activity (U) was defined as the amount of enzyme that converts 1 nmol nitrocefin per minute. Specific activity (U/mg) was expressed as nanomoles of nitrocefin hydrolyzed per minute per milligram of protein.

### Real-time quantitative PCR (qRT-PCR).

The preparation of DNA-free RNA and reverse transcription were carried out as described previously (35). qRT-PCRs were performed using the ABI StepOnePlus real-time PCR system. The primers used for qRT-PCR are listed in Table S7. The relative expression levels were determined by normalizing transcription to 16S rRNA and calculated using the threshold cycle (ΔΔ*C_T_*) method (57). Each assay was performed at least three times by independent experiments.

### N-phenylnaphtylamine (NPN) uptake assay.

The logarithmically grown cells were harvested, washed with 5 mM HEPES buffer (pH 7.2), and adjusted to an optical density at 450 nm (OD_450_) of 0.5 using the same buffer. The 96-well microtiter plate wells were supplemented with 100-μL bacterial suspensions and 15 μM NPN. After a 5-min incubation, the fluorescence was monitored using a fluorescence spectrophotometer (Tecan Infinite 200 PRO) at excitation and emission wavelengths of 355 nm and 402 nm, respectively. Fluorescence is emitted by NPN only after it partitions into the membrane; therefore, a greater emission of fluorescence represents greater outer membrane permeability to NPN.

### Data availability.

The RNA-seq data have been deposited in GenBank BioSample accessions SAMN25290492 for S. maltophilia KJ and SAMN30672914 for KJΔOmpA_299-356_.
