# Supplementary material for: σP-NagA-L1/L2 Regulatory Circuit Involved in ΔompA299-356-Mediated Increase in β-Lactam Susceptibility in Stenotrophomonas maltophilia
Source: Microbiol Spectr. 2022 Nov 9;10(6):e02797-22. doi: 10.1128/spectrum.02797-22 (PMC9769791; doi:10.1128/spectrum.02797-22)
Supplement: Supplemental file 1 — Fig. S1 to S3 and Tables S1 to S4, S6, and S7. Download spectrum.02797-22-s0001.pdf, PDF file, 5.3 MB [file spectrum.02797-22-s0001.pdf]

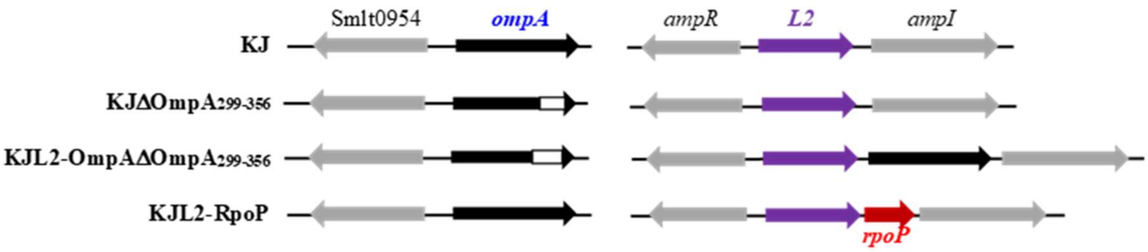

Fig. S1. Genomic organization of KJ and its derived constructs. The orientation of the gene is indicated by the arrow. The white box indicates the deleted region of *ompA* gene. Genes are color-coded: black, *ompA*; purple, *L2*; red, *rpoP*.

```
A. baumannii     1    M--KLSRIALATMLVAAPLAAANAGVTVTPLLLGYTFQDSQHNNGGKDGNLTNSPELQDD  58
S. maltophilia   1    MNKKILTAALLGGLAFAQAASAQE------------FDDRWYLTGSAGFNFQDSDRLTND  48

A. baumannii     59   L-FVGAALGIELTPWLGFEAEYNQVKGDVDGASAGAEYKQKQINGNFYVTSDLITKNYDS  117
S. maltophilia   49   APFVTLGLGKFISPNWSLDGELNYQNPNFDA--------NKDMNWSQYGVSLDLRRHFIK  100

A. baumannii     118  KIK---PYVLLGAGHYKYD--FDGVNRGTRGTSEEGTLGNAGVGAFWRLNDALSLRTEAR  172
S. maltophilia   101  EGRGWNPYLLAGLGYQKSEEEYNPISGGLADRKDGNFAAKVGVGLQTTFEKRVAVRAEV-  159

A. baumannii     173  ATYNAD-------------EEFWNYTALAGLNVVLGGHLKPAAPVVEVAPVEPTPVAPQP  219
S. maltophilia   160  -AYRADFDDQSVNPKRAGNDESWFGDVLASVGVVI-----PLGPAPVAAAPAPAPVAPSC  213

A. baumannii     220  QELTED----------------------------LNMELR-VFFDTNKSNIKDQYKPEIA  250
S. maltophilia   214  ADLDDDGDGVNNCDDKCPNSQPGQTIGPDGCPVPVSIDLKGVNFDFDKSNLRPDAVAILS  273

A. baumannii     251  KVAEKLSEYPNATARIEGHTDNTGPRKLNERLSLARANSVKSALVNEYNVDASRL-STQG  309
S. maltophilia   274  EATEILKRYPDLRVEVAGHTDSKGTDAYNQKLSERRATAVYNYLTKN-GVDAGRLVGPIG  332

A. baumannii     310  FAWDQPIADNKTK------EGRAMNRRVFATITGSRTVVVQPGQEAAAPAAAQ  356
S. maltophilia   333  YGESRPIAPNTNPDGSDNPEGRAKNRRTELNVQN                     366
```

Fig. S2. OmpA proteins alignment of *A. baumannii* and *S. maltophilia*. The conserved amino acid residues were marked in gray. The key residues involved in PG interaction were marked in red.

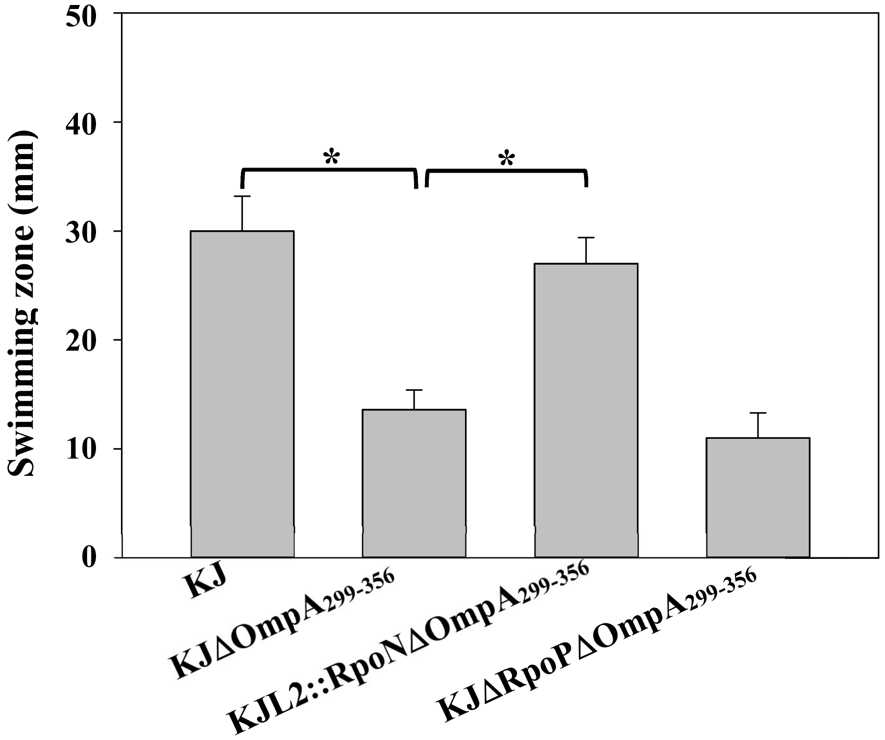

Fig. S3. The swimming motility of wild-type KJ and its derived *ompA* mutants. Overnighted cultured bacterial cells were inoculated into fresh LB broth and then grown for 5 h. Five microliters of bacterial cell aliquot were inoculated into swimming agar. After a 48-h incubation at 37°C for 48 h, the swimming zones were recorded. Data are the means from three independent experiments. Error bars indicate the standard deviations for three triplicate samples. *, $P < 0.01$, significance calculated by Student's *t* test.

Table S1. LC-MS/MS analysis of the band A protein spot (Fig. 1, 37-kDa)

>Smlt0955 (OmpA)

MNKKILTAAL LGGLAFAQAA SAQEFDDRWY LTGSAGFNFQ DSDRLTNDAP FVTLGLGKFI SPNWSLDGEL NYQNPNFDAN
KDMNWSQYGV SLDLRRHFIK EGRGWNPYLL AGLGYQKSEE EYNPISGGLA DRKDGNFAAK VGVGLQTTFE KRVAVRAEVA
YRADFDDQSV NPKRAGNDES WFGDVLASVG VVIPLGPAPV AAAPAPAPVA PSCADLDDDG DGVNNCDDKC PNSQPGQTIG
PDGCPVPVSI DLKGVNFDFD KSNLRPDAVA ILSEATEILK RYPDLRVEVA GHTDSKGTDA YNQKLSERRA TAVYNYLTKN
GVDAGRLVGP IGYGESRPIA PNTNPDGSDN PEGRAKNRRT ELNVQN

#	zBP	File	z	dM	MH+	Xcorr	dCn	Sp	RSp	Ions	Reference	( ) Sequence
651	-----	03149	3	0.0	2683.3	7.25	0.54	4735	1	47/88	gi|190010910	(K) FISPNWSLDGELNYQNPNFDANK
735	-----	03472	3	0.0	2196.3	7.24	0.64	4756	1	47/76	gi|190010910	(K) SNLRPDAVAILSEATEILKR
764	-----	03631	3	0.0	2196.3	6.94	0.67	4320	1	45/76	gi|190010910	(K) SNLRPDAVAILSEATEILKR
721	-----	03402	3	0.0	2196.3	6.92	0.65	4410	1	45/76	gi|190010910	(K) SNLRPDAVAILSEATEILKR
752	-----	03545	3	0.0	2196.3	6.81	0.64	5233	1	48/76	gi|190010910	(K) SNLRPDAVAILSEATEILKR
635	-----	03074	3	0.0	2683.3	6.66	0.52	4335	1	45/88	gi|190010910	(K) FISPNWSLDGELNYQNPNFDANK
846	-----	04105	3	0.0	2683.2	6.64	0.58	3346	1	42/88	gi|190010910	(K) FISPNWSLDGELNYQNPNFDANK
694	-----	03303	3	0.0	2683.3	6.42	0.58	2860	1	40/88	gi|190010910	(K) FISPNWSLDGELNYQNPNFDANK
790	-----	03774	3	0.0	2196.3	6.40	0.63	3616	1	43/76	gi|190010910	(K) SNLRPDAVAILSEATEILKR
777	-----	03703	3	0.0	2196.3	6.40	0.64	3855	1	43/76	gi|190010910	(K) SNLRPDAVAILSEATEILKR
671	-----	03228	3	0.0	2683.3	6.39	0.53	2848	1	42/88	gi|190010910	(K) FISPNWSLDGELNYQNPNFDANK
809	-----	03899	3	0.0	2962.6	6.37	0.69	3120	1	44/104	gi|190010910	(K) GVNFDFDKSNLRPDAVAILSEATEILK
828	-----	04013	3	0.0	2962.6	6.37	0.61	3701	1	44/104	gi|190010910	(K) GVNFDFDKSNLRPDAVAILSEATEILK
844	-----	04090	3	0.0	2962.6	6.22	0.60	3623	1	45/104	gi|190010910	(K) GVNFDFDKSNLRPDAVAILSEATEILK
865	-----	04187	3	0.0	2962.6	6.22	0.65	4165	1	46/104	gi|190010910	(K) GVNFDFDKSNLRPDAVAILSEATEILK
398	-----	02027	3	0.0	2879.4	6.08	0.63	2742	1	47/108	gi|190010910	(R) LVGPIGYGESRPIAPNTNPDGSDNPEGR
618	-----	03010	3	0.0	2196.3	6.01	0.65	3356	1	44/76	gi|190010910	(K) SNLRPDAVAILSEATEILKR
513	-----	02573	3	0.0	2879.4	5.89	0.67	1547	1	40/108	gi|190010910	(R) LVGPIGYGESRPIAPNTNPDGSDNPEGR
741	-----	03500	3	0.0	2879.4	5.88	0.65	2499	1	47/108	gi|190010910	(R) LVGPIGYGESRPIAPNTNPDGSDNPEGR
686	-----	03266	3	0.0	2879.4	5.87	0.63	2382	1	45/108	gi|190010910	(R) LVGPIGYGESRPIAPNTNPDGSDNPEGR
920	-----	04481	3	0.0	2683.2	5.80	0.56	3764	1	45/88	gi|190010910	(K) FISPNWSLDGELNYQNPNFDANK
796	-----	03818	3	0.0	2962.6	5.79	0.62	3235	1	43/104	gi|190010910	(K) GVNFDFDKSNLRPDAVAILSEATEILK
564	-----	02801	3	0.0	2879.4	5.77	0.65	2049	1	44/108	gi|190010910	(R) LVGPIGYGESRPIAPNTNPDGSDNPEGR
747	-----	03524	3	0.0	2962.6	5.74	0.61	3380	1	44/104	gi|190010910	(K) GVNFDFDKSNLRPDAVAILSEATEILK
362	-----	01871	3	0.0	2879.4	5.73	0.58	2576	1	46/108	gi|190010910	(R) LVGPIGYGESRPIAPNTNPDGSDNPEGR
412	-----	02105	3	0.0	2879.4	5.71	0.60	1901	1	43/108	gi|190010910	(R) LVGPIGYGESRPIAPNTNPDGSDNPEGR
533	-----	02651	3	0.0	2879.4	5.69	0.62	1768	1	43/108	gi|190010910	(R) LVGPIGYGESRPIAPNTNPDGSDNPEGR
887	-----	04316	3	0.0	2879.4	5.67	0.66	2406	1	46/108	gi|190010910	(R) LVGPIGYGESRPIAPNTNPDGSDNPEGR
480	-----	02417	3	0.0	2879.4	5.65	0.66	2680	1	49/108	gi|190010910	(R) LVGPIGYGESRPIAPNTNPDGSDNPEGR
600	-----	02957	3	0.0	2879.4	5.62	0.64	1929	1	44/108	gi|190010910	(R) LVGPIGYGESRPIAPNTNPDGSDNPEGR
772	-----	03679	3	0.0	2683.3	5.62	0.59	3702	1	43/88	gi|190010910	(K) FISPNWSLDGELNYQNPNFDANK
724	-----	03421	3	0.0	2879.4	5.56	0.66	1971	1	44/108	gi|190010910	(R) LVGPIGYGESRPIAPNTNPDGSDNPEGR
894	-----	04365	3	0.0	2962.6	5.56	0.62	1974	1	40/104	gi|190010910	(K) GVNFDFDKSNLRPDAVAILSEATEILK
464	-----	02339	3	0.0	2879.4	5.54	0.65	2246	1	44/108	gi|190010910	(R) LVGPIGYGESRPIAPNTNPDGSDNPEGR
452	-----	02261	3	0.0	2879.4	5.52	0.65	2042	1	45/108	gi|190010910	(R) LVGPIGYGESRPIAPNTNPDGSDNPEGR
708	-----	03369	2	0.0	2040.2	5.46	0.65	2392	1	26/36	gi|190010910	(K) SNLRPDAVAILSEATEILK
583	-----	02879	3	0.0	2879.4	5.44	0.62	2143	1	46/108	gi|190010910	(R) LVGPIGYGESRPIAPNTNPDGSDNPEGR
750	-----	03538	2	0.0	2040.2	5.43	0.70	2549	1	26/36	gi|190010910	(K) SNLRPDAVAILSEATEILK
495	-----	02495	3	0.0	2879.4	5.43	0.63	2112	1	45/108	gi|190010910	(R) LVGPIGYGESRPIAPNTNPDGSDNPEGR
648	-----	03136	2	0.0	2040.2	5.36	0.75	2231	1	25/36	gi|190010910	(K) SNLRPDAVAILSEATEILK
817	-----	03946	3	0.0	2683.3	5.36	0.59	2796	1	42/88	gi|190010910	(K) FISPNWSLDGELNYQNPNFDANK
875	-----	04249	3	0.0	2683.3	5.32	0.66	3259	1	40/88	gi|190010910	(K) FISPNWSLDGELNYQNPNFDANK
433	-----	02183	3	0.0	2879.4	5.30	0.61	1748	1	43/108	gi|190010910	(R) LVGPIGYGESRPIAPNTNPDGSDNPEGR
861	-----	04175	3	0.0	2683.2	5.30	0.63	2376	1	39/88	gi|190010910	(K) FISPNWSLDGELNYQNPNFDANK
661	-----	03189	3	0.0	2879.4	5.27	0.63	2333	1	46/108	gi|190010910	(R) LVGPIGYGESRPIAPNTNPDGSDNPEGR
546	-----	02723	3	0.0	2879.4	5.25	0.62	2138	1	43/108	gi|190010910	(R) LVGPIGYGESRPIAPNTNPDGSDNPEGR
753	-----	03553	2	0.0	1863.9	5.23	0.71	2572	1	25/30	gi|190010910	(R) WYLTGSAGFNFQDSDR
416	-----	02119	4	0.0	2632.3	5.18	0.53	2238	1	57/138	gi|190010910	(K) CPNSQPGQTIGPDGCPVPVSIDLK
778	-----	03706	3	0.0	2962.6	5.17	0.63	3116	1	42/104	gi|190010910	(K) GVNFDFDKSNLRPDAVAILSEATEILK
918	-----	04467	3	0.0	2879.4	5.15	0.58	1984	1	42/108	gi|190010910	(R) LVGPIGYGESRPIAPNTNPDGSDNPEGR
762	-----	03625	2	0.0	2040.2	5.15	0.72	2398	1	26/36	gi|190010910	(K) SNLRPDAVAILSEATEILK
802	-----	03843	2	0.0	2040.2	5.14	0.73	2030	1	25/36	gi|190010910	(K) SNLRPDAVAILSEATEILK
806	-----	03868	2	0.0	1863.9	5.12	0.68	2248	1	24/30	gi|190010910	(R) WYLTGSAGFNFQDSDR
872	-----	04227	2	0.0	1863.8	5.12	0.76	2403	1	25/30	gi|190010910	(R) WYLTGSAGFNFQDSDR
701	-----	03342	3	0.0	2879.4	5.11	0.57	2345	1	44/108	gi|190010910	(R) LVGPIGYGESRPIAPNTNPDGSDNPEGR
771	-----	03677	3	0.0	2879.4	5.07	0.67	2725	1	48/108	gi|190010910	(R) LVGPIGYGESRPIAPNTNPDGSDNPEGR
732	-----	03461	2	0.0	1863.9	5.06	0.72	2461	1	25/30	gi|190010910	(R) WYLTGSAGFNFQDSDR
860	-----	04174	3	0.0	2879.4	5.05	0.64	2191	1	44/108	gi|190010910	(R) LVGPIGYGESRPIAPNTNPDGSDNPEGR
789	-----	03772	2	0.0	2040.2	5.00	0.72	2637	1	27/36	gi|190010910	(K) SNLRPDAVAILSEATEILK
736	-----	03479	4	0.0	2196.3	4.99	0.60	4102	1	57/114	gi|190010910	(K) SNLRPDAVAILSEATEILKR
632	-----	03061	2	0.0	1579.9	4.96	0.47	2071	1	23/26	gi|190010910	(R) GWNPYLLAGLGYQK
765	-----	03643	2	0.0	1863.9	4.96	0.74	2468	1	25/30	gi|190010910	(R) WYLTGSAGFNFQDSDR
767	-----	03649	4	0.0	2196.3	4.93	0.64	3326	1	52/114	gi|190010910	(K) SNLRPDAVAILSEATEILKR
705	-----	03356	2	0.0	1579.9	4.93	0.48	2113	1	23/26	gi|190010910	(R) GWNPYLLAGLGYQK
647	-----	03131	2	0.0	1579.8	4.92	0.48	2171	1	23/26	gi|190010910	(R) GWNPYLLAGLGYQK
901	-----	04392	3	0.0	2879.4	4.91	0.62	2643	1	47/108	gi|190010910	(R) LVGPIGYGESRPIAPNTNPDGSDNPEGR
375	-----	01927	2	0.0	1636.8	4.89	0.64	2105	1	24/28	gi|190010910	(K) SEEEYNPISGGLADR
776	-----	03702	2	0.0	2040.2	4.87	0.69	2196	1	25/36	gi|190010910	(K) SNLRPDAVAILSEATEILK
361	-----	01858	2	0.0	1636.8	4.86	0.67	2123	1	24/28	gi|190010910	(K) SEEEYNPISGGLADR
689	-----	03277	2	0.0	1579.9	4.86	0.47	2143	1	23/26	gi|190010910	(R) GWNPYLLAGLGYQK
831	-----	04027	3	0.0	2683.2	4.85	0.54	2066	1	35/88	gi|190010910	(K) FISPNWSLDGELNYQNPNFDANK
731	-----	03460	3	0.0	2683.3	4.84	0.58	2152	1	36/88	gi|190010910	(K) FISPNWSLDGELNYQNPNFDANK
446	-----	02236	2	0.0	1636.8	4.83	0.66	2093	1	24/28	gi|190010910	(K) SEEEYNPISGGLADR
666	-----	03201	2	0.0	1579.9	4.82	0.48	1880	1	22/26	gi|190010910	(R) GWNPYLLAGLGYQK
793	-----	03794	2	0.0	1863.9	4.81	0.70	2611	1	25/30	gi|190010910	(R) WYLTGSAGFNFQDSDR
745	-----	03511	2	0.0	1579.9	4.79	0.50	1708	1	21/26	gi|190010910	(R) GWNPYLLAGLGYQK
785	-----	03762	3	0.0	2879.4	4.79	0.60	2353	1	46/108	gi|190010910	(R) LVGPIGYGESRPIAPNTNPDGSDNPEGR
723	-----	03420	2	0.0	1683.8	4.77	0.58	2089	1	22/26	gi|190010910	(K) DMNWSQYGVSLDLR
424	-----	02157	2	0.0	1636.8	4.73	0.67	2080	1	24/28	gi|190010910	(K) SEEEYNPISGGLADR
885	-----	04305	2	0.0	1863.8	4.72	0.61	1801	1	23/30	gi|190010910	(R) WYLTGSAGFNFQDSDR
845	-----	04103	3	0.0	2879.4	4.72	0.59	1861	1	42/108	gi|190010910	(R) LVGPIGYGESRPIAPNTNPDGSDNPEGR
389	-----	02002	2	0.0	1636.8	4.71	0.69	2167	1	24/28	gi|190010910	(K) SEEEYNPISGGLADR

781	-----	03722	4	0.0	2196.3	4.69	0.60	4500	1	58/114	gi|190010910	(K)SNLRPDAVAILSEATEILKR
799	-----	03825	4	0.0	2196.3	4.69	0.60	4382	1	56/114	gi|190010910	(K)SNLRPDAVAILSEATEILKR
402	-----	02047	4	0.0	2632.3	4.68	0.54	2253	1	56/138	gi|190010910	(K)CPNSQPGQTIGPDGCPVPVSIDLK
816	-----	03945	3	0.0	2879.4	4.63	0.56	2428	1	43/108	gi|190010910	(R)LVGPIGYGESRPIAPNTNPDGSDNPEGR
873	-----	04246	3	0.0	2879.4	4.63	0.67	1938	1	41/108	gi|190010910	(R)LVGPIGYGESRPIAPNTNPDGSDNPEGR
720	-----	03401	4	0.0	2196.3	4.62	0.59	3467	1	53/114	gi|190010910	(K)SNLRPDAVAILSEATEILKR
853	-----	04144	2	0.0	1579.8	4.60	0.52	1964	1	22/26	gi|190010910	(R)GWNPYLLAGLGYQK
835	-----	04050	2	0.0	2040.1	4.56	0.61	1611	1	25/36	gi|190010910	(K)SNLRPDAVAILSEATEILK
406	-----	02079	2	0.0	1636.8	4.56	0.66	2138	1	24/28	gi|190010910	(K)SEEEYNPISGGLADR
650	-----	03143	3	0.0	1579.8	4.49	0.45	2162	1	31/52	gi|190010910	(R)GWNPYLLAGLGYQK
739	-----	03498	2	0.0	1683.8	4.46	0.57	2282	1	23/26	gi|190010910	(K)DMNWSQYGVSLDLR
712	-----	03382	3	0.0	2683.3	4.45	0.55	1693	1	35/88	gi|190010910	(K)FISPNWSLDGELNYQNPNFDANK
594	-----	02931	2	0.0	1699.8	4.44	0.61	2319	1	23/26	gi|190010910	(K)DM*NWSQYGVSLDLR
331	-----	01743	2	0.0	1764.9	4.43	0.61	1628	1	24/30	gi|190010910	(K)SEEEYNPISGGLADRK
633	-----	03068	3	0.0	1579.8	4.43	0.48	2266	1	33/52	gi|190010910	(R)GWNPYLLAGLGYQK
342	-----	01783	2	0.0	1636.8	4.43	0.58	1762	1	22/28	gi|190010910	(K)SEEEYNPISGGLADR
286	-----	01587	2	0.0	1764.9	4.42	0.58	1899	1	26/30	gi|190010910	(K)SEEEYNPISGGLADRK
264	-----	01511	2	0.0	1764.9	4.41	0.59	1771	1	24/30	gi|190010910	(K)SEEEYNPISGGLADRK
904	-----	04406	2	0.0	1863.8	4.41	0.70	1704	1	22/30	gi|190010910	(R)WYLTGSAGFNFQDSDR
727	-----	03433	2	0.0	1579.8	4.41	0.60	1625	1	21/26	gi|190010910	(R)GWNPYLLAGLGYQK
676	-----	03240	2	0.0	1683.8	4.40	0.56	2059	1	22/26	gi|190010910	(K)DMNWSQYGVSLDLR
640	-----	03111	3	0.0	2879.4	4.38	0.62	1731	1	42/108	gi|190010910	(R)LVGPIGYGESRPIAPNTNPDGSDNPEGR
698	-----	03316	2	0.0	1683.8	4.37	0.59	2064	1	22/26	gi|190010910	(K)DMNWSQYGVSLDLR
821	-----	03967	3	0.0	2196.2	4.36	0.59	2428	1	39/76	gi|190010910	(K)SNLRPDAVAILSEATEILKR
921	-----	04496	2	0.0	1863.8	4.35	0.70	2376	1	25/30	gi|190010910	(R)WYLTGSAGFNFQDSDR
577	-----	02853	2	0.0	1699.8	4.32	0.60	2341	1	23/26	gi|190010910	(K)DM*NWSQYGVSLDLR
305	-----	01665	2	0.0	1764.9	4.31	0.63	1867	1	25/30	gi|190010910	(K)SEEEYNPISGGLADRK
129	-----	00979	3	0.0	1919.9	4.30	0.62	1812	1	37/68	gi|190010910	(R)VEVAGHTDSKGTDAYNQK
101	-----	00841	2	0.0	1919.9	4.29	0.68	1510	1	25/34	gi|190010910	(R)VEVAGHTDSKGTDAYNQK
654	-----	03162	2	0.0	1683.8	4.29	0.59	2292	1	23/26	gi|190010910	(K)DMNWSQYGVSLDLR
823	-----	03992	2	0.0	1579.8	4.27	0.56	1797	1	22/26	gi|190010910	(R)GWNPYLLAGLGYQK
566	-----	02808	2	0.0	1445.8	4.27	0.49	1149	1	22/26	gi|190010910	(R)LTNDAPFVTLGLGK
794	-----	03798	2	0.0	1636.8	4.24	0.62	1628	1	21/28	gi|190010910	(K)SEEEYNPISGGLADR
839	-----	04062	2	0.0	1579.8	4.23	0.52	1992	1	22/26	gi|190010910	(R)GWNPYLLAGLGYQK
183	-----	01201	2	0.0	1750.8	4.22	0.63	1718	1	25/32	gi|190010910	(R)PIAPNTNPDGSDNPEGR
163	-----	01123	2	0.0	1750.8	4.20	0.72	1683	1	23/32	gi|190010910	(R)PIAPNTNPDGSDNPEGR
784	-----	03749	3	0.0	2683.3	4.19	0.51	2939	1	40/88	gi|190010910	(K)FISPNWSLDGELNYQNPNFDANK
550	-----	02736	2	0.0	1445.8	4.15	0.58	1396	1	24/26	gi|190010910	(R)LTNDAPFVTLGLGK
569	-----	02821	2	0.0	1445.8	4.14	0.56	1391	1	23/26	gi|190010910	(R)LTNDAPFVTLGLGK
586	-----	02892	2	0.0	1445.8	4.14	0.55	1351	1	23/26	gi|190010910	(R)LTNDAPFVTLGLGK
854	-----	04145	2	0.0	1683.8	4.13	0.61	2067	1	21/26	gi|190010910	(K)DMNWSQYGVSLDLR
900	-----	04391	3	0.0	2040.1	4.12	0.60	1274	1	32/72	gi|190010910	(K)SNLRPDAVAILSEATEILK
491	-----	02470	2	0.0	1636.8	4.12	0.61	1409	1	20/28	gi|190010910	(K)SEEEYNPISGGLADR
733	-----	03466	2	0.0	1445.8	4.11	0.58	1193	1	22/26	gi|190010910	(R)LTNDAPFVTLGLGK
365	-----	01878	4	0.0	2879.4	4.08	0.39	1907	1	57/162	gi|190010910	(R)LVGPIGYGESRPIAPNTNPDGSDNPEGR
798	-----	03821	2	0.0	1579.8	4.08	0.50	1764	1	21/26	gi|190010910	(R)GWNPYLLAGLGYQK
184	-----	01203	2	0.0	1919.9	4.08	0.73	1116	1	23/34	gi|190010910	(R)VEVAGHTDSKGTDAYNQK
749	-----	03537	3	0.0	2040.2	4.07	0.52	1496	1	32/72	gi|190010910	(K)SNLRPDAVAILSEATEILK
830	-----	04026	3	0.0	2879.4	4.05	0.62	1505	1	42/108	gi|190010910	(R)LVGPIGYGESRPIAPNTNPDGSDNPEGR
758	-----	03583	3	0.0	2879.4	4.04	0.62	2131	1	40/108	gi|190010910	(R)LVGPIGYGESRPIAPNTNPDGSDNPEGR
933	-----	04532	2	0.0	1683.8	4.04	0.60	2119	1	22/26	gi|190010910	(K)DMNWSQYGVSLDLR
242	-----	01433	2	0.0	1750.8	4.04	0.65	1241	1	22/32	gi|190010910	(R)PIAPNTNPDGSDNPEGR
86	-----	00772	2	0.0	1919.9	4.04	0.70	1533	1	25/34	gi|190010910	(R)VEVAGHTDSKGTDAYNQK
755	-----	03573	2	0.0	1683.8	4.03	0.66	1916	1	22/26	gi|190010910	(K)DMNWSQYGVSLDLR
761	-----	03618	3	0.0	2040.2	4.02	0.55	1417	1	32/72	gi|190010910	(K)SNLRPDAVAILSEATEILK
114	-----	00919	2	0.0	1919.9	4.00	0.67	1490	1	26/34	gi|190010910	(R)VEVAGHTDSKGTDAYNQK
69	-----	00702	2	0.0	1919.9	3.99	0.71	1214	1	24/34	gi|190010910	(R)VEVAGHTDSKGTDAYNQK
881	-----	04282	4	0.0	2962.6	3.98	0.53	1927	1	48/156	gi|190010910	(K)GVNFDFDKSNLRPDAVAILSEATEILK
787	-----	03766	3	0.0	2040.2	3.97	0.55	1298	1	29/72	gi|190010910	(K)SNLRPDAVAILSEATEILK
768	-----	03665	2	0.0	1683.8	3.97	0.60	1833	1	21/26	gi|190010910	(K)DMNWSQYGVSLDLR
263	-----	01510	2	0.0	1750.8	3.95	0.58	1260	1	22/32	gi|190010910	(R)PIAPNTNPDGSDNPEGR
684	-----	03255	2	0.0	1699.8	3.93	0.56	1515	1	20/26	gi|190010910	(K)DM*NWSQYGVSLDLR
757	-----	03582	2	0.0	1579.8	3.92	0.50	1530	1	21/26	gi|190010910	(R)GWNPYLLAGLGYQK
628	-----	03039	3	0.0	2879.4	3.92	0.55	1460	1	40/108	gi|190010910	(R)LVGPIGYGESRPIAPNTNPDGSDNPEGR
604	-----	02964	2	0.0	1445.8	3.91	0.58	760	1	19/26	gi|190010910	(R)LTNDAPFVTLGLGK
135	-----	00997	3	0.0	2405.2	3.90	0.57	1349	1	36/84	gi|190010910	(R)VEVAGHTDSKGTDAYNQKLSER
289	-----	01591	2	0.0	1750.8	3.88	0.68	1171	1	22/32	gi|190010910	(R)PIAPNTNPDGSDNPEGR
617	-----	03009	2	0.0	1699.8	3.88	0.64	1673	1	21/26	gi|190010910	(K)DM*NWSQYGVSLDLR
536	-----	02667	2	0.0	1445.8	3.87	0.60	1137	1	22/26	gi|190010910	(R)LTNDAPFVTLGLGK
201	-----	01279	2	0.0	1750.8	3.85	0.66	1284	1	22/32	gi|190010910	(R)PIAPNTNPDGSDNPEGR
780	-----	03717	2	0.0	1863.9	3.81	0.64	1255	1	22/30	gi|190010910	(R)WYLTGSAGFNFQDSDR
100	-----	00836	3	0.0	1919.9	3.81	0.51	2046	1	38/68	gi|190010910	(R)VEVAGHTDSKGTDAYNQK
848	-----	04110	3	0.0	2040.1	3.80	0.60	1122	1	30/72	gi|190010910	(K)SNLRPDAVAILSEATEILK
932	-----	04531	3	0.0	2040.1	3.80	0.53	1042	1	29/72	gi|190010910	(K)SNLRPDAVAILSEATEILK
459	-----	02313	2	0.0	1636.8	3.77	0.64	1443	1	21/28	gi|190010910	(K)SEEEYNPISGGLADR
506	-----	02548	2	0.0	1636.8	3.77	0.64	1787	1	21/28	gi|190010910	(K)SEEEYNPISGGLADR
659	-----	03182	2	0.0	1445.8	3.75	0.46	741	1	19/26	gi|190010910	(R)LTNDAPFVTLGLGK
639	-----	03110	2	0.0	1445.8	3.72	0.52	931	1	20/26	gi|190010910	(R)LTNDAPFVTLGLGK
893	-----	04345	3	0.0	2196.2	3.72	0.67	1650	1	33/76	gi|190010910	(K)SNLRPDAVAILSEATEILKR
220	-----	01355	2	0.0	1750.8	3.71	0.67	850	1	19/32	gi|190010910	(R)PIAPNTNPDGSDNPEGR
476	-----	02392	2	0.0	1636.8	3.71	0.36	1143	1	23/28	gi|190010910	(K)SEEEYNPISGGLADR
442	-----	02222	4	0.0	2632.3	3.70	0.48	2042	1	53/138	gi|190010910	(K)CPNSQPGQTIGPDGCPVPVSIDLK
774	-----	03696	3	0.0	2040.2	3.69	0.41	1251	1	32/72	gi|190010910	(K)SNLRPDAVAILSEATEILK
451	-----	02251	3	0.0	2632.3	3.68	0.57	646	1	36/92	gi|190010910	(K)CPNSQPGQTIGPDGCPVPVSIDLK
824	-----	03997	3	0.0	2040.1	3.65	0.57	1098	1	30/72	gi|190010910	(K)SNLRPDAVAILSEATEILK
754	-----	03557	4	0.0	2196.3	3.64	0.44	1867	1	45/114	gi|190010910	(K)SNLRPDAVAILSEATEILKR
83	-----	00766	3	0.0	1919.9	3.64	0.58	1325	1	33/68	gi|190010910	(R)VEVAGHTDSKGTDAYNQK
535	-----	02659	2	0.0	1178.7	3.63	0.45	1306	1	17/20	gi|190010910	(K)VGVGLQTTFEK

276	-----	01555	3	0.0	1299.7	**3.21**	**0.51**	**1281**	**1**	25/40	gi|190010910	(R)RATAVYNYLTK
822	-----	03991	2	0.0	1683.8	**3.20**	**0.46**	**1045**	**1**	18/26	gi|190010910	(K)DMNWSQYGVSLDLR
287	-----	01588	2	0.0	1334.8	**3.20**	**0.49**	**712**	**1**	14/22	gi|190010910	(K)VGVGLQTTFEKR
327	-----	01731	2	0.0	1334.8	**3.19**	**0.46**	**849**	**1**	15/22	gi|190010910	(K)VGVGLQTTFEKR
18	-----	00431	2	0.0	1042.5	**3.18**	**0.62**	**1363**	**1**	16/18	gi|190010910	(R)VEVAGHTDSK
913	-----	04440	2	0.0	1579.8	**3.17**	**0.47**	**1249**	**1**	17/26	gi|190010910	(R)GWNPYLLAGLGYQK
870	-----	04219	2	0.0	1178.6	**3.17**	**0.47**	**1344**	**1**	17/20	gi|190010910	(K)VGVGLQTTFEK
228	-----	01377	2	0.0	1299.7	**3.17**	**0.55**	**1136**	**1**	16/20	gi|190010910	(R)RATAVYNYLTK
814	-----	03926	3	0.0	2040.2	**3.17**	**0.52**	**1507**	**1**	34/72	gi|190010910	(K)SNLRPDAVAILSEATEILK
797	-----	03820	2	0.0	1683.8	**3.15**	**0.60**	**1351**	**1**	20/26	gi|190010910	(K)DMNWSQYGVSLDLR
655	-----	03164	2	0.0	1699.8	**3.15**	**0.48**	**606**	**1**	17/26	gi|190010910	(K)DM*NWSQYGVSLDLR
882	-----	04292	2	0.0	1579.8	**3.14**	**0.57**	**1926**	**1**	20/26	gi|190010910	(R)GWNPYLLAGLGYQK
346	-----	01795	2	0.0	1750.8	**3.14**	**0.69**	**933**	**1**	20/32	gi|190010910	(R)PIAPNTNPDGSDNPEGR
303	-----	01661	2	0.0	1334.8	**3.14**	**0.40**	**813**	**1**	15/22	gi|190010910	(K)VGVGLQTTFEKR
500	-----	02506	2	0.0	1299.7	**3.13**	**0.36**	**1153**	**1**	17/20	gi|190010910	(R)RATAVYNYLTK
314	-----	01685	2	0.0	1143.6	**3.12**	**0.65**	**952**	**1**	15/18	gi|190010910	(R)ATAVYNYLTK
164	-----	01124	3	0.0	1919.9	**3.12**	**0.44**	**1433**	**1**	32/68	gi|190010910	(R)VEVAGHTDSKGTDAYNQK
397	-----	02023	2	0.0	1334.8	**3.11**	**0.40**	**885**	**1**	16/22	gi|190010910	(K)VGVGLQTTFEKR
871	-----	04225	2	0.0	1683.8	**3.09**	**0.59**	**1000**	**1**	17/26	gi|190010910	(K)DMNWSQYGVSLDLR
868	-----	04213	4	0.0	2962.6	**3.08**	**0.35**	**1840**	**1**	49/156	gi|190010910	(K)GVNFDFDKSNLRPDAVAILSEATEILK
230	-----	01382	3	0.0	1919.9	**3.08**	**0.46**	**912**	**1**	29/68	gi|190010910	(R)VEVAGHTDSKGTDAYNQK
167	-----	01143	2	0.0	1235.6	**3.08**	**0.44**	**1624**	**1**	18/20	gi|190010910	(R)ADFDDQSVNPK
393	-----	02006	2	0.0	1764.9	**3.07**	**0.33**	**667**	**1**	18/30	gi|190010910	(K)SEEEYNPISGGLADRK
381	-----	01971	2	0.0	1299.7	**3.06**	**0.60**	**1210**	**1**	17/20	gi|190010910	(R)RATAVYNYLTK
273	-----	01544	3	0.0	1686.9	**3.06**	**0.56**	**776**	**1**	30/56	gi|190010910	(R)YPDLRVEVAGHTDSK
791	-----	03782	2	0.0	1299.7	**3.05**	**0.50**	**834**	**1**	16/20	gi|190010910	(R)RATAVYNYLTK
165	-----	01125	2	0.0	1919.9	**3.05**	**0.60**	**1001**	**1**	23/34	gi|190010910	(R)VEVAGHTDSKGTDAYNQK
40	-----	00574	2	0.0	1042.5	**3.03**	**0.63**	**942**	**1**	15/18	gi|190010910	(R)VEVAGHTDSK
527	-----	02629	2	0.0	1636.8	**3.03**	**0.54**	**646**	**1**	18/28	gi|190010910	(K)SEEEYNPISGGLADR
468	-----	02363	2	0.0	1334.8	**3.03**	**0.34**	**909**	**1**	15/22	gi|190010910	(K)VGVGLQTTFEKR
320	-----	01698	4	0.0	3078.6	**3.03**	**0.51**	**1226**	**1**	59/174	gi|190010910	(R)LVGPIGYGESRPIAPNTNPDGSDNPEGRAK
427	-----	02170	2	0.0	1334.8	**3.02**	**0.38**	**869**	**1**	16/22	gi|190010910	(K)VGVGLQTTFEKR
434	-----	02186	2	0.0	1299.7	**3.02**	**0.47**	**1312**	**1**	18/20	gi|190010910	(R)RATAVYNYLTK
718	-----	03396	2	0.0	1445.8	**3.02**	**0.53**	**868**	**1**	19/26	gi|190010910	(R)LTNDAPFVTLGLGK
855	-----	04146	2	0.0	1863.8	**3.02**	**0.56**	**1027**	**1**	19/30	gi|190010910	(R)WYLTGSAGFNFQDSDR
90	-----	00793	2	0.0	1042.5	**3.01**	**0.66**	**884**	**1**	15/18	gi|190010910	(R)VEVAGHTDSK
288	-----	01589	2	0.0	1299.7	**2.99**	**0.46**	**1377**	**1**	18/20	gi|190010910	(R)RATAVYNYLTK
576	-----	02850	2	0.0	1334.8	**2.99**	**0.38**	**707**	**1**	14/22	gi|190010910	(K)VGVGLQTTFEKR
619	-----	03011	2	0.0	1334.8	**2.99**	**0.35**	**1229**	**1**	18/22	gi|190010910	(K)VGVGLQTTFEKR
910	-----	04423	4	0.0	2962.6	**3.41**	**0.50**	**1587**	**1**	46/156	gi|190010910	(K)GVNFDFDKSNLRPDAVAILSEATEILK
355	-----	01826	2	0.0	1178.7	**3.40**	**0.47**	**1369**	**1**	17/20	gi|190010910	(K)VGVGLQTTFEK
914	-----	04447	2	0.0	1178.6	**3.40**	**0.45**	**1332**	**1**	17/20	gi|190010910	(K)VGVGLQTTFEK
384	-----	01983	2	0.0	1178.7	**3.40**	**0.51**	**1262**	**1**	17/20	gi|190010910	(K)VGVGLQTTFEK
548	-----	02730	2	0.0	1178.7	**3.40**	**0.45**	**1361**	**1**	17/20	gi|190010910	(K)VGVGLQTTFEK
836	-----	04052	2	0.0	1636.7	**3.38**	**0.65**	**1156**	**1**	19/28	gi|190010910	(K)SEEEYNPISGGLADR
206	-----	01299	2	0.0	1235.6	**3.37**	**0.45**	**1357**	**1**	17/20	gi|190010910	(R)ADFDDQSVNPK
485	-----	02434	3	0.0	2632.3	**3.37**	**0.54**	**676**	**1**	35/92	gi|190010910	(K)CPNSQPGQTIGPDGCPVPVSIDLK
685	-----	03256	2	0.0	1636.8	**3.37**	**0.54**	**1198**	**1**	20/28	gi|190010910	(K)SEEEYNPISGGLADR
769	-----	03670	2	0.0	1178.7	**3.36**	**0.51**	**1397**	**1**	17/20	gi|190010910	(K)VGVGLQTTFEK
876	-----	04253	3	0.0	2040.1	**3.35**	**0.58**	**1258**	**1**	32/72	gi|190010910	(K)SNLRPDAVAILSEATEILK
95	-----	00817	2	0.0	1381.7	**3.35**	**0.59**	**847**	**1**	17/22	gi|190010910	(K)GTDAYNQKLSER
744	-----	03505	2	0.0	1178.7	**3.35**	**0.43**	**1341**	**1**	17/20	gi|190010910	(K)VGVGLQTTFEK
470	-----	02372	2	0.0	1178.7	**3.33**	**0.44**	**1330**	**1**	17/20	gi|190010910	(K)VGVGLQTTFEK
348	-----	01801	2	0.0	1334.8	**3.33**	**0.40**	**1000**	**1**	16/22	gi|190010910	(K)VGVGLQTTFEKR
704	-----	03349	2	0.0	1178.7	**3.33**	**0.47**	**1307**	**1**	17/20	gi|190010910	(K)VGVGLQTTFEK
730	-----	03448	2	0.0	1699.8	**3.32**	**0.44**	**1197**	**1**	20/26	gi|190010910	(K)DM*NWSQYGVSLDLR
825	-----	03998	2	0.0	1178.6	**3.32**	**0.53**	**1393**	**1**	17/20	gi|190010910	(K)VGVGLQTTFEK
247	-----	01452	2	0.0	1235.6	**3.31**	**0.39**	**950**	**1**	16/20	gi|190010910	(R)ADFDDQSVNPK
488	-----	02450	2	0.0	1178.7	**3.30**	**0.44**	**1395**	**1**	17/20	gi|190010910	(K)VGVGLQTTFEK
455	-----	02294	2	0.0	1178.7	**3.30**	**0.45**	**1266**	**1**	17/20	gi|190010910	(K)VGVGLQTTFEK
266	-----	01517	2	0.0	1334.8	**3.28**	**0.38**	**883**	**1**	16/22	gi|190010910	(K)VGVGLQTTFEKR
883	-----	04297	2	0.0	1178.6	**3.28**	**0.50**	**1303**	**1**	17/20	gi|190010910	(K)VGVGLQTTFEK
627	-----	03037	2	0.0	1445.8	**3.27**	**0.54**	**866**	**1**	19/26	gi|190010910	(R)LTNDAPFVTLGLGK
856	-----	04149	2	0.0	1178.6	**3.26**	**0.46**	**1353**	**1**	17/20	gi|190010910	(K)VGVGLQTTFEK
190	-----	01228	3	0.0	1919.9	**3.25**	**0.41**	**1136**	**1**	30/68	gi|190010910	(R)VEVAGHTDSKGTDAYNQK
807	-----	03876	4	0.0	2196.3	**3.24**	**0.49**	**2560**	**1**	46/114	gi|190010910	(K)SNLRPDAVAILSEATEILKR
567	-----	02809	2	0.0	1178.7	**3.23**	**0.50**	**1342**	**1**	17/20	gi|190010910	(K)VGVGLQTTFEK
461	-----	02328	3	0.0	2632.3	**3.23**	**0.52**	**470**	**1**	35/92	gi|190010910	(K)CPNSQPGQTIGPDGCPVPVSIDLK
338	-----	01759	3	0.0	2468.2	**3.22**	**0.53**	**2078**	**1**	35/88	gi|190010910	(K)SEEEYNPISGGLADRKDGNFAAK
687	-----	03271	2	0.0	1178.7	**3.22**	**0.47**	**1302**	**1**	17/20	gi|190010910	(K)VGVGLQTTFEK
811	-----	03909	2	0.0	1579.8	**3.21**	**0.44**	**1068**	**1**	18/26	gi|190010910	(R)GWNPYLLAGLGYQK
276	-----	01555	3	0.0	1299.7	**3.21**	**0.51**	**1281**	**1**	25/40	gi|190010910	(R)RATAVYNYLTK
822	-----	03991	2	0.0	1683.8	**3.20**	**0.46**	**1045**	**1**	18/26	gi|190010910	(K)DMNWSQYGVSLDLR
287	-----	01588	2	0.0	1334.8	**3.20**	**0.49**	**712**	**1**	14/22	gi|190010910	(K)VGVGLQTTFEKR
327	-----	01731	2	0.0	1334.8	**3.19**	**0.46**	**849**	**1**	15/22	gi|190010910	(K)VGVGLQTTFEKR
18	-----	00431	2	0.0	1042.5	**3.18**	**0.62**	**1363**	**1**	16/18	gi|190010910	(R)VEVAGHTDSK
913	-----	04440	2	0.0	1579.8	**3.17**	**0.47**	**1249**	**1**	17/26	gi|190010910	(R)GWNPYLLAGLGYQK
870	-----	04219	2	0.0	1178.6	**3.17**	**0.47**	**1344**	**1**	17/20	gi|190010910	(K)VGVGLQTTFEK
228	-----	01377	2	0.0	1299.7	**3.17**	**0.55**	**1136**	**1**	16/20	gi|190010910	(R)RATAVYNYLTK
814	-----	03926	3	0.0	2040.2	**3.17**	**0.52**	**1507**	**1**	34/72	gi|190010910	(K)SNLRPDAVAILSEATEILK
797	-----	03820	2	0.0	1683.8	**3.15**	**0.60**	**1351**	**1**	20/26	gi|190010910	(K)DMNWSQYGVSLDLR
655	-----	03164	2	0.0	1699.8	**3.15**	**0.48**	**606**	**1**	17/26	gi|190010910	(K)DM*NWSQYGVSLDLR
882	-----	04292	2	0.0	1579.8	**3.14**	**0.57**	**1926**	**1**	20/26	gi|190010910	(R)GWNPYLLAGLGYQK
346	-----	01795	2	0.0	1750.8	**3.14**	**0.69**	**933**	**1**	20/32	gi|190010910	(R)PIAPNTNPDGSDNPEGR
303	-----	01661	2	0.0	1334.8	**3.14**	**0.40**	**813**	**1**	15/22	gi|190010910	(K)VGVGLQTTFEKR
500	-----	02506	2	0.0	1299.7	**3.13**	**0.36**	**1153**	**1**	17/20	gi|190010910	(R)RATAVYNYLTK
314	-----	01685	2	0.0	1143.6	**3.12**	**0.65**	**952**	**1**	15/18	gi|190010910	(R)ATAVYNYLTK
164	-----	01124	3	0.0	1919.9	**3.12**	**0.44**	**1433**	**1**	32/68	gi|190010910	(R)VEVAGHTDSKGTDAYNQK
397	-----	02023	2	0.0	1334.8	**3.11**	**0.40**	**885**	**1**	16/22	gi|190010910	(K)VGVGLQTTFEKR
871	-----	04225	2	0.0	1683.8	**3.09**	**0.59**	**1000**	**1**	17/26	gi|190010910	(K)DMNWSQYGVSLDLR
868	-----	04213	4	0.0	2962.6	**3.08**	**0.35**	**1840**	**1**	49/156	gi|190010910	(K)GVNFDFDKSNLRPDAVAILSEATEILK
230	-----	01382	3	0.0	1919.9	**3.08**	**0.46**	**912**	**1**	29/68	gi|190010910	(R)VEVAGHTDSKGTDAYNQK
167	-----	01143	2	0.0	1235.6	**3.08**	**0.44**	**1624**	**1**	18/20	gi|190010910	(R)ADFDDQSVNPK
393	-----	02006	2	0.0	1764.9	**3.07**	**0.33**	**667**	**1**	18/30	gi|190010910	(K)SEEEYNPISGGLADRK
381	-----	01971	2	0.0	1299.7	**3.06**	**0.60**	**1210**	**1**	17/20	gi|190010910	(R)RATAVYNYLTK
273	-----	01544	3	0.0	1686.9	**3.06**	**0.56**	**776**	**1**	30/56	gi|190010910	(R)YPDLRVEVAGHTDSK

791	-----	03782	2	0.0	1299.7	3.05	0.50	834	1	16/20	gi|190010910	(R)RATAVYNYLTK
165	-----	01125	2	0.0	1919.9	3.05	0.60	1001	1	23/34	gi|190010910	(R)VEVAGHTDSKGTDAYNQK
40	-----	00574	2	0.0	1042.5	3.03	0.63	942	1	15/18	gi|190010910	(R)VEVAGHTDSK
527	-----	02629	2	0.0	1636.8	3.03	0.54	646	1	18/28	gi|190010910	(K)SEEEYNPISGGLADR
468	-----	02363	2	0.0	1334.8	3.03	0.34	909	1	15/22	gi|190010910	(K)VGVGLQTTFEKR
320	-----	01698	4	0.0	3078.6	3.03	0.51	1226	1	59/174	gi|190010910	(R)LVGPIGYGESRPIAPNTNPDGSDNPEGRAK
427	-----	02170	2	0.0	1334.8	3.02	0.38	869	1	16/22	gi|190010910	(K)VGVGLQTTFEKR
434	-----	02186	2	0.0	1299.7	3.02	0.47	1312	1	18/20	gi|190010910	(R)RATAVYNYLTK
718	-----	03396	2	0.0	1445.8	3.02	0.53	868	1	19/26	gi|190010910	(R)LTNDAPFVTLGLGK
855	-----	04146	2	0.0	1863.8	3.02	0.56	1027	1	19/30	gi|190010910	(R)WYLTGSAGFNFQDSDR
90	-----	00793	2	0.0	1042.5	3.01	0.66	884	1	15/18	gi|190010910	(R)VEVAGHTDSK
288	-----	01589	2	0.0	1299.7	2.99	0.46	1377	1	18/20	gi|190010910	(R)RATAVYNYLTK
576	-----	02850	2	0.0	1334.8	2.99	0.38	707	1	14/22	gi|190010910	(K)VGVGLQTTFEKR
619	-----	03011	2	0.0	1334.8	2.99	0.35	1229	1	18/22	gi|190010910	(K)VGVGLQTTFEKR
842	-----	04076	3	0.0	2040.1	2.99	0.46	1360	1	32/72	gi|190010910	(K)SNLRPDAVAILSEATEILK
149	-----	01067	2	0.0	1235.6	2.98	0.52	1692	1	18/20	gi|190010910	(R)ADFDDQSVNPK
378	-----	01948	2	0.0	1334.8	2.98	0.38	872	1	15/22	gi|190010910	(K)VGVGLQTTFEKR
903	-----	04405	3	0.0	2683.2	2.96	0.38	705	14	25/88	gi|190010910	(K)FISPNWSLDGELNYQNPNFDANK
726	-----	03427	2	0.0	1178.7	2.95	0.47	1340	1	17/20	gi|190010910	(K)VGVGLQTTFEK
615	-----	02999	3	0.0	2683.3	2.95	0.43	650	14	25/88	gi|190010910	(K)FISPNWSLDGELNYQNPNFDANK
132	-----	00991	2	0.0	1235.6	2.95	0.49	1368	1	16/20	gi|190010910	(R)ADFDDQSVNPK
151	-----	01069	3	0.0	1750.8	2.95	0.33	1032	1	31/64	gi|190010910	(R)PIAPNTNPDGSDNPEGR
339	-----	01763	2	0.0	1143.6	2.94	0.64	888	1	15/18	gi|190010910	(R)ATAVYNYLTK
306	-----	01666	2	0.0	1299.7	2.93	0.47	1445	1	18/20	gi|190010910	(R)RATAVYNYLTK
27	-----	00504	2	0.0	1042.5	2.92	0.63	1066	1	16/18	gi|190010910	(R)VEVAGHTDSK
57	-----	00643	2	0.0	1042.5	2.92	0.64	894	1	15/18	gi|190010910	(R)VEVAGHTDSK
629	-----	03045	2	0.0	1178.7	2.91	0.48	1329	1	17/20	gi|190010910	(K)VGVGLQTTFEK
841	-----	04075	4	0.0	2962.6	2.91	0.35	1444	1	47/156	gi|190010910	(K)GVNFDFDKSNLRPDAVAILSEATEILK
895	-----	04366	2	0.0	1579.8	2.91	0.67	1122	1	17/26	gi|190010910	(R)GWNPYLLAGLGYQK
283	-----	01581	2	0.0	1147.6	2.90	0.67	1232	1	19/20	gi|190010910	(R)LVGPIGYGESR
411	-----	02101	2	0.0	1334.8	2.90	0.28	981	1	16/22	gi|190010910	(K)VGVGLQTTFEKR
849	-----	04112	3	3.0	2193.3	2.90	0.43	1477	1	29/76	gi|190010910	(K)SNLRPDAVAILSEATEILKR
329	-----	01738	2	0.0	1299.7	2.89	0.50	1238	1	17/20	gi|190010910	(R)RATAVYNYLTK
788	-----	03770	2	0.0	1334.8	2.89	0.31	624	1	14/22	gi|190010910	(K)VGVGLQTTFEKR
783	-----	03745	2	0.0	1178.7	2.89	0.50	1390	1	17/20	gi|190010910	(K)VGVGLQTTFEK
756	-----	03579	2	0.0	1178.7	2.89	0.45	1338	1	16/20	gi|190010910	(K)VGVGLQTTFEK
408	-----	02092	3	0.0	2632.3	2.87	0.48	449	1	34/92	gi|190010910	(K)CPNSQPGQTIGPDGCPVPVSIDLK
683	-----	03254	2	0.0	1334.8	2.86	0.34	795	1	15/22	gi|190010910	(K)VGVGLQTTFEKR
388	-----	01996	2	0.0	1143.6	2.85	0.67	875	1	15/18	gi|190010910	(R)ATAVYNYLTK
269	-----	01531	2	0.0	1235.6	2.85	0.24	1122	2	16/20	gi|190010910	(R)ADFDDQSVNPK
265	-----	01516	2	0.0	1299.7	2.85	0.43	1362	1	18/20	gi|190010910	(R)RATAVYNYLTK
245	-----	01445	2	0.0	1299.7	2.83	0.50	1406	1	18/20	gi|190010910	(R)RATAVYNYLTK
107	-----	00876	2	0.0	1042.5	2.83	0.46	891	1	15/18	gi|190010910	(R)VEVAGHTDSK
349	-----	01807	2	0.0	1299.7	2.83	0.49	1059	1	16/20	gi|190010910	(R)RATAVYNYLTK
751	-----	03544	4	0.0	2040.2	2.82	0.24	1789	5	41/108	gi|190010910	(K)SNLRPDAVAILSEATEILK
484	-----	02433	2	0.0	1334.8	2.81	0.47	731	1	14/22	gi|190010910	(K)VGVGLQTTFEKR
414	-----	02113	2	0.0	1299.7	2.80	0.53	1323	1	18/20	gi|190010910	(R)RATAVYNYLTK
358	-----	01840	2	0.0	1143.6	2.80	0.56	868	1	15/18	gi|190010910	(R)ATAVYNYLTK
773	-----	03687	2	0.0	1334.8	2.79	0.48	637	1	15/22	gi|190010910	(K)VGVGLQTTFEKR
693	-----	03301	2	0.0	1042.5	2.79	0.54	949	1	15/18	gi|190010910	(R)VEVAGHTDSK
590	-----	02907	2	0.0	1839.9	2.78	0.41	230	4	14/28	gi|190010910	(K)DMNWSQYGVSLDLRR
801	-----	03831	4	0.0	2962.6	2.78	0.24	2127	1	50/156	gi|190010910	(K)GVNFDFDKSNLRPDAVAILSEATEILK
878	-----	04265	2	0.0	1334.7	2.78	0.47	804	1	16/22	gi|190010910	(K)VGVGLQTTFEKR
908	-----	04419	2	0.0	1334.7	2.77	0.38	587	1	14/22	gi|190010910	(K)VGVGLQTTFEKR
490	-----	02463	2	0.0	1143.6	2.75	0.47	997	1	15/18	gi|190010910	(R)ATAVYNYLTK
852	-----	04142	4	0.0	2962.6	2.74	0.13	2316	1	52/156	gi|190010910	(K)GVNFDFDKSNLRPDAVAILSEATEILK
118	-----	00933	3	0.0	1949.9	2.73	0.45	533	1	28/72	gi|190010910	(R)PIAPNTNPDGSDNPEGRAK
14	-----	00382	2	0.0	886.5	2.72	0.31	872	2	12/12	gi|190010910	(R)HFIKEGR
226	-----	01375	2	0.0	1235.6	2.72	0.21	544	2	14/20	gi|190010910	(R)ADFDDQSVNPK
374	-----	01912	2	0.0	1143.6	2.71	0.62	1024	1	16/18	gi|190010910	(R)ATAVYNYLTK
636	-----	03085	2	0.0	1699.8	2.70	0.42	308	2	14/26	gi|190010910	(K)DM*NWSQYGVSLDLR
400	-----	02040	2	0.0	1299.7	2.69	0.49	1405	1	18/20	gi|190010910	(R)RATAVYNYLTK
734	-----	03469	2	0.0	1334.8	2.69	0.37	425	2	13/22	gi|190010910	(K)VGVGLQTTFEKR
144	-----	01031	2	0.0	1042.5	2.66	0.52	1053	1	15/18	gi|190010910	(R)VEVAGHTDSK
291	-----	01602	2	0.0	1686.9	2.66	0.61	565	1	18/28	gi|190010910	(R)YPDLRVEVAGHTDSK
453	-----	02281	2	0.0	1143.6	2.64	0.62	961	1	15/18	gi|190010910	(R)ATAVYNYLTK
833	-----	04029	2	0.0	1334.7	2.63	0.31	616	1	14/22	gi|190010910	(K)VGVGLQTTFEKR
595	-----	02940	2	0.0	1334.8	2.61	0.35	813	1	16/22	gi|190010910	(K)VGVGLQTTFEKR
552	-----	02747	2	0.0	1334.8	2.61	0.32	564	3	14/22	gi|190010910	(K)VGVGLQTTFEKR
125	-----	00954	2	0.0	1042.5	2.60	0.54	928	1	15/18	gi|190010910	(R)VEVAGHTDSK
766	-----	03645	2	0.0	1636.8	2.60	0.51	325	1	16/28	gi|190010910	(K)SEEEYNPISGGLADR
298	-----	01640	2	0.0	1299.7	2.59	0.39	1437	1	18/20	gi|190010910	(R)RATAVYNYLTK
531	-----	02641	2	0.0	1334.8	2.59	0.42	750	1	16/22	gi|190010910	(K)VGVGLQTTFEKR
665	-----	03196	3	0.0	2040.2	2.58	0.46	1170	3	30/72	gi|190010910	(K)SNLRPDAVAILSEATEILK
467	-----	02360	2	0.0	1143.6	2.57	0.61	979	1	15/18	gi|190010910	(R)ATAVYNYLTK
925	-----	04513	3	0.0	2196.2	2.57	0.40	1547	1	36/76	gi|190010910	(K)SNLRPDAVAILSEATEILKR
922	-----	04500	2	0.0	1334.7	2.56	0.38	643	2	14/22	gi|190010910	(K)VGVGLQTTFEKR
899	-----	04389	3	0.0	1299.7	2.55	0.30	1114	3	23/40	gi|190010910	(R)RATAVYNYLTK
810	-----	03904	2	0.0	1683.8	2.55	0.53	587	1	15/26	gi|190010910	(K)DMNWSQYGVSLDLR
795	-----	03801	4	0.0	2196.3	2.53	0.51	1728	3	41/114	gi|190010910	(K)SNLRPDAVAILSEATEILKR
930	-----	04526	2	0.0	1299.7	2.53	0.51	932	1	16/20	gi|190010910	(R)RATAVYNYLTK
803	-----	03852	3	0.0	2879.4	2.53	0.41	834	1	32/108	gi|190010910	(R)LVGPIGYGESRPIAPNTNPDGSDNPEGR
492	-----	02476	3	0.0	1855.9	2.52	0.49	635	1	24/56	gi|190010910	(K)DM*NWSQYGVSLDLRR
800	-----	03827	2	0.0	1178.7	2.51	0.42	945	1	15/20	gi|190010910	(K)VGVGLQTTFEK
819	-----	03953	2	0.0	1334.7	2.50	0.42	460	3	12/22	gi|190010910	(K)VGVGLQTTFEKR
74	-----	00717	2	0.0	1042.5	2.50	0.59	947	1	15/18	gi|190010910	(R)VEVAGHTDSK
890	-----	04336	2	0.0	1299.7	2.49	0.43	1352	1	18/20	gi|190010910	(R)RATAVYNYLTK
523	-----	02596	2	0.0	1299.7	2.49	0.61	1212	1	18/20	gi|190010910	(R)RATAVYNYLTK
863	-----	04178	2	0.0	1299.7	2.47	0.40	781	1	15/20	gi|190010910	(R)RATAVYNYLTK
438	-----	02205	4	0.0	1686.9	2.44	0.47	1352	3	38/84	gi|190010910	(R)YPDLRVEVAGHTDSK
703	-----	03346	2	0.0	1636.8	2.42	0.53	600	1	18/28	gi|190010910	(K)SEEEYNPISGGLADR
907	-----	04413	3	0.0	1686.8	2.42	0.29	614	1	26/56	gi|190010910	(R)YPDLRVEVAGHTDSK
879	-----	04266	2	0.0	1042.5	2.42	0.52	778	1	14/18	gi|190010910	(R)VEVAGHTDSK
431	-----	02179	2	0.0	1143.6	2.39	0.51	1139	1	16/18	gi|190010910	(R)ATAVYNYLTK
134	-----	00995	2	0.0	1391.7	2.39	0.48	734	2	16/22	gi|190010910	(R)ADFDDQSVNPKR
25	-----	00467	2	0.0	886.5	2.39	0.36	694	2	10/12	gi|190010910	(R)HFIKEGR
10	-----	00350	2	0.0	1042.5	2.37	0.53	1039	1	15/18	gi|190010910	(R)VEVAGHTDSK

826	-----	04003	4	0.0	2962.6	2.36	0.11	2195	1	51/156	gi|190010910	(K)GVNFDFDKSNLRPDAVAILSEATEILK
317	-----	01689	2	0.0	1235.6	2.35	0.38	955	1	16/20	gi|190010910	(R)ADFDDQSVNPK
850	-----	04114	2	0.0	1042.5	2.35	0.49	605	1	13/18	gi|190010910	(R)VEVAGHTDSK
906	-----	04412	2	0.0	1042.5	2.34	0.64	745	1	14/18	gi|190010910	(R)VEVAGHTDSK
657	-----	03175	2	0.0	1334.8	2.33	0.33	634	1	15/22	gi|190010910	(K)VGVGLQTTFEKR
612	-----	02993	2	0.0	1042.5	2.33	0.50	703	1	14/18	gi|190010910	(R)VEVAGHTDSK
371	-----	01893	2	0.0	1299.7	2.31	0.50	475	3	12/20	gi|190010910	(R)RATAVYNYLTK
570	-----	02827	2	0.0	1839.9	2.30	0.43	311	1	16/28	gi|190010910	(K)DMNWSQYGVSLDLRR
573	-----	02838	2	0.0	1299.7	2.29	0.51	498	2	13/20	gi|190010910	(R)RATAVYNYLTK
166	-----	01133	2	0.0	1042.5	2.28	0.58	757	1	14/18	gi|190010910	(R)VEVAGHTDSK
924	-----	04512	2	0.0	1042.5	2.28	0.51	694	1	13/18	gi|190010910	(R)VEVAGHTDSK
325	-----	01721	2	0.0	1750.8	2.28	0.43	669	1	18/32	gi|190010910	(R)PIAPNTNPDGSDNPEGR
241	-----	01430	2	0.0	1042.5	2.27	0.28	652	1	12/18	gi|190010910	(R)VEVAGHTDSK
782	-----	03740	2	0.0	1683.8	2.25	0.59	915	1	16/26	gi|190010910	(K)DMNWSQYGVSLDLR
877	-----	04255	2	0.0	1299.7	2.25	0.42	726	1	15/20	gi|190010910	(R)RATAVYNYLTK
537	-----	02671	3	0.0	1445.8	2.25	0.41	1066	3	24/52	gi|190010910	(R)LTNDAPFVTLGLGK
905	-----	04407	2	0.0	1299.7	2.24	0.44	489	1	13/20	gi|190010910	(R)RATAVYNYLTK
888	-----	04323	3	0.0	2040.1	2.23	0.28	1150	1	29/72	gi|190010910	(K)SNLRPDAVAILSEATEILK
37	-----	00552	2	0.0	886.5	2.22	0.35	919	1	12/12	gi|190010910	(R)HFIKEGR
892	-----	04344	2	0.0	1334.7	2.21	0.33	255	2	12/22	gi|190010910	(K)VGVGLQTTFEKR
818	-----	03948	2	0.0	1299.7	2.20	0.38	765	1	13/20	gi|190010910	(R)RATAVYNYLTK
293	-----	01609	4	0.0	1686.9	2.19	0.40	1275	2	37/84	gi|190010910	(R)YPDLRVEVAGHTDSK
11	-----	00358	2	0.0	896.4	2.17	0.51	633	1	12/14	gi|190010910	(K)GTDAYNQK
611	-----	02990	3	0.0	1839.9	2.16	0.41	790	2	25/56	gi|190010910	(K)DMNWSQYGVSLDLRR
775	-----	03700	2	0.0	1299.7	2.14	0.32	585	2	13/20	gi|190010910	(R)RATAVYNYLTK
19	-----	00432	2	0.0	896.4	2.14	0.42	587	1	12/14	gi|190010910	(K)GTDAYNQK
847	-----	04106	2	0.0	1334.7	2.14	0.34	413	6	11/22	gi|190010910	(K)VGVGLQTTFEKR
345	-----	01791	2	0.0	1235.6	2.13	0.26	699	1	14/20	gi|190010910	(R)ADFDDQSVNPK
4	-----	00289	2	0.0	896.4	2.11	0.51	692	1	13/14	gi|190010910	(K)GTDAYNQK
813	-----	03922	2	0.0	1143.6	2.11	0.50	578	2	13/18	gi|190010910	(R)ATAVYNYLTK
363	-----	01873	2	0.0	1334.8	2.11	0.47	850	1	15/22	gi|190010910	(K)VGVGLQTTFEKR
267	-----	01519	3	0.0	1764.9	2.09	0.26	564	1	27/60	gi|190010910	(K)SEEEYNPISGGLADRK
556	-----	02764	2	0.0	1299.7	2.08	0.51	599	1	13/20	gi|190010910	(R)RATAVYNYLTK
5	-----	00300	2	0.0	850.4	2.07	0.01	675	1	14/14	gi|190010910	(R)KDGNFAAK
34	-----	00539	2	0.0	850.4	2.06	0.13	438	1	11/14	gi|190010910	(R)KDGNFAAK
680	-----	03247	3	0.0	1683.8	2.04	0.14	1093	7	26/52	gi|190010910	(K)DMNWSQYGVSLDLR
637	-----	03099	2	0.0	1334.8	2.03	0.41	502	1	14/22	gi|190010910	(K)VGVGLQTTFEKR
106	-----	00874	2	0.0	973.5	2.01	0.30	637	1	11/14	gi|190010910	(R)RTELNVQN
554	-----	02757	3	0.0	1839.9	2.01	0.47	387	8	22/56	gi|190010910	(K)DMNWSQYGVSLDLRR
92	-----	00802	2	0.0	973.5	2.00	0.31	623	1	11/14	gi|190010910	(R)RTELNVQN
443	-----	02231	2	0.0	1235.6	1.98	0.32	898	1	16/20	gi|190010910	(R)ADFDDQSVNPK
383	-----	01973	3	0.0	1299.7	1.98	0.23	824	9	18/40	gi|190010910	(R)RATAVYNYLTK
347	-----	01800	2	0.0	941.5	1.97	0.52	477	1	12/14	gi|190010910	(K)GVNFDFDK
21	-----	00455	2	0.0	850.4	1.97	0.04	465	1	12/14	gi|190010910	(R)KDGNFAAK
909	-----	04420	3	0.0	2196.2	1.96	0.01	656	6	27/76	gi|190010910	(K)SNLRPDAVAILSEATEILKR
31	-----	00525	3	0.0	1042.5	1.95	0.35	731	1	20/36	gi|190010910	(R)VEVAGHTDSK
808	-----	03877	3	0.0	2196.3	1.95	0.39	534	16	24/76	gi|190010910	(K)SNLRPDAVAILSEATEILKR
820	-----	03960	4	0.0	2196.2	1.94	0.26	1649	10	39/114	gi|190010910	(K)SNLRPDAVAILSEATEILKR
678	-----	03242	2	0.0	1299.7	1.91	0.46	715	1	14/20	gi|190010910	(R)RATAVYNYLTK
559	-----	02782	2	0.0	819.5	1.90	0.11	256	19	8/10	gi|190010910	(K)RYPDLR
834	-----	04045	4	0.0	1686.8	1.89	0.28	919	16	32/84	gi|190010910	(R)YPDLRVEVAGHTDSK
931	-----	04529	3	0.0	1686.8	1.87	0.34	412	4	23/56	gi|190010910	(R)YPDLRVEVAGHTDSK
123	-----	00952	2	0.0	973.5	1.86	0.31	712	1	12/14	gi|190010910	(R)RTELNVQN
199	-----	01274	2	0.0	973.5	1.85	0.14	580	2	11/14	gi|190010910	(R)RTELNVQN
714	-----	03390	2	0.0	941.4	1.84	0.23	489	11	10/14	gi|190010910	(K)GVNFDFDK
108	-----	00901	3	0.0	1042.5	1.83	0.34	658	1	20/36	gi|190010910	(R)VEVAGHTDSK
396	-----	02022	2	0.0	941.5	1.83	0.38	509	2	12/14	gi|190010910	(K)GVNFDFDK
545	-----	02720	2	0.0	1042.5	1.82	0.45	439	7	10/18	gi|190010910	(R)VEVAGHTDSK
866	-----	04195	2	0.0	1042.5	1.82	0.50	582	1	12/18	gi|190010910	(R)VEVAGHTDSK
81	-----	00756	3	0.0	1381.7	1.82	0.45	474	7	20/44	gi|190010910	(K)GTDAYNQKLSER
45	-----	00597	3	0.0	1042.5	1.82	0.31	877	1	22/36	gi|190010910	(R)VEVAGHTDSK
20	-----	00444	3	0.0	1042.5	1.81	0.38	724	1	22/36	gi|190010910	(R)VEVAGHTDSK
471	-----	02374	2	0.0	819.5	1.81	0.15	256	19	8/10	gi|190010910	(K)RYPDLR
579	-----	02861	2	0.0	819.5	1.81	0.16	299	14	9/10	gi|190010910	(K)RYPDLR
76	-----	00729	2	0.0	973.5	1.80	0.29	716	1	12/14	gi|190010910	(R)RTELNVQN
616	-----	03006	4	0.0	2196.3	1.80	0.01	1636	1	43/114	gi|190010910	(K)SNLRPDAVAILSEATEILKR
838	-----	04058	3	0.0	1686.8	1.79	0.26	490	7	23/56	gi|190010910	(R)YPDLRVEVAGHTDSK
563	-----	02799	2	0.0	1042.5	1.78	0.42	655	2	12/18	gi|190010910	(R)VEVAGHTDSK
688	-----	03272	4	0.0	2196.3	1.77	0.12	1724	1	43/114	gi|190010910	(K)SNLRPDAVAILSEATEILKR
540	-----	02679	2	0.0	1299.7	1.75	0.23	687	1	15/20	gi|190010910	(R)RATAVYNYLTK
923	-----	04502	4	0.0	1686.8	1.74	0.24	746	14	32/84	gi|190010910	(R)YPDLRVEVAGHTDSK
667	-----	03211	2	0.0	1042.5	1.74	0.25	651	1	14/18	gi|190010910	(R)VEVAGHTDSK
508	-----	02556	3	0.0	1299.7	1.73	0.08	805	3	21/40	gi|190010910	(R)RATAVYNYLTK
591	-----	02912	3	0.0	1839.9	1.72	0.28	333	19	21/56	gi|190010910	(K)DMNWSQYGVSLDLRR
143	-----	01028	2	0.0	973.5	1.72	0.26	626	1	11/14	gi|190010910	(R)RTELNVQN
571	-----	02834	3	0.0	1839.9	1.72	0.36	306	15	21/56	gi|190010910	(K)DMNWSQYGVSLDLRR
290	-----	01594	3	0.0	1764.9	1.69	0.46	554	1	29/60	gi|190010910	(K)SEEEYNPISGGLADRK
867	-----	04197	4	0.0	1686.8	1.69	0.28	751	20	31/84	gi|190010910	(R)YPDLRVEVAGHTDSK
394	-----	02011	4	0.0	1686.9	1.68	0.11	1611	3	38/84	gi|190010910	(R)YPDLRVEVAGHTDSK
181	-----	01195	2	0.0	973.5	1.68	0.21	749	2	12/14	gi|190010910	(R)RTELNVQN
179	-----	01184	3	0.0	1042.5	1.67	0.26	802	1	21/36	gi|190010910	(R)VEVAGHTDSK
804	-----	03862	2	0.0	1334.8	1.67	0.33	176	7	11/22	gi|190010910	(K)VGVGLQTTFEKR
874	-----	04248	2	0.0	1445.8	1.66	0.42	613	1	16/26	gi|190010910	(R)LTNDAPFVTLGLGK
311	-----	01673	3	0.0	1764.9	1.65	0.44	570	1	29/60	gi|190010910	(K)SEEEYNPISGGLADRK
409	-----	02099	2	0.0	941.5	1.64	0.40	368	7	9/14	gi|190010910	(K)GVNFDFDK
880	-----	04276	2	0.0	941.4	1.63	0.19	331	8	9/14	gi|190010910	(K)GVNFDFDK
544	-----	02706	2	0.0	819.5	1.62	0.16	255	18	8/10	gi|190010910	(K)RYPDLR
71	-----	00708	2	0.0	708.4	1.61	0.25	523	3	9/10	gi|190010910	(R)AEVAYR
697	-----	03312	2	0.0	941.4	1.60	0.08	464	6	11/14	gi|190010910	(K)GVNFDFDK

430	-----	02178	2	0.0	941.4	1.59	**0.42**	353	4	11/14	gi|190010910		(K)GVNFDFDK
77	-----	00742	4	0.0	1919.9	1.59	**0.37**	435	4	37/102	gi|190010910		(R)VEVAGHTDSKGTDAYNQK
518	-----	02582	3	0.0	1042.5	1.59	0.05	**710**	**2**	17/36	gi|190010910		(R)VEVAGHTDSK
420	-----	02140	2	0.0	819.5	1.58	**0.17**	268	19	8/10	gi|190010910		(K)RYPDLR
829	-----	04021	2	0.0	1299.7	1.57	**0.27**	363	8	11/20	gi|190010910		(R)RATAVYNYLTK
38	-----	00561	2	0.0	708.4	1.55	**0.37**	465	**1**	9/10	gi|190010910		(R)AEVAYR
902	-----	04399	2	0.0	1235.6	1.54	**0.37**	361	8	13/20	gi|190010910		(R)ADFDDQSVNPK
380	-----	01967	2	0.0	1686.9	1.54	**0.33**	466	**1**	16/28	gi|190010910		(R)YPDLRVEVAGHTDSK
837	-----	04056	2	0.0	941.4	1.53	**0.22**	462	**2**	12/14	gi|190010910		(K)GVNFDFDK
54	-----	00631	2	0.0	708.4	1.52	**0.38**	470	**1**	9/10	gi|190010910		(R)AEVAYR
886	-----	04314	2	0.0	973.5	1.50	0.10	**710**	**1**	12/14	gi|190010910		(R)RTELNVQN
815	-----	03932	2	0.0	1042.5	1.50	**0.40**	386	8	10/18	gi|190010910		(R)VEVAGHTDSK
124	-----	00953	3	0.0	1391.7	1.49	**0.41**	495	4	21/44	gi|190010910		(R)ADFDDQSVNPKR
700	-----	03337	2	0.0	973.5	1.48	0.07	473	10	9/14	gi|190010910		(R)RTELNVQN
673	-----	03235	2	0.0	941.4	1.46	**0.24**	331	7	10/14	gi|190010910		(K)GVNFDFDK
748	-----	03528	2	0.0	1299.7	1.44	**0.24**	533	**1**	13/20	gi|190010910		(R)RATAVYNYLTK
827	-----	04010	2	0.0	1235.6	1.41	**0.25**	355	9	11/20	gi|190010910		(R)ADFDDQSVNPK
675	-----	03238	2	0.0	973.5	1.41	**0.20**	**714**	**1**	11/14	gi|190010910		(R)RTELNVQN
326	-----	01728	2	0.0	941.4	1.39	0.02	339	11	10/14	gi|190010910		(K)GVNFDFDK
96	-----	00823	3	0.0	1042.5	1.39	**0.26**	582	**1**	19/36	gi|190010910		(R)VEVAGHTDSK
78	-----	00743	3	0.0	1042.5	1.39	0.09	499	5	17/36	gi|190010910		(R)VEVAGHTDSK
911	-----	04424	2	0.0	941.4	1.38	0.05	241	12	8/14	gi|190010910		(K)GVNFDFDK
162	-----	01117	2	0.0	973.5	1.37	0.03	**639**	17	11/14	gi|190010910		(R)RTELNVQN
148	-----	01066	2	0.0	663.4	1.33	0.08	408	**2**	7/8	gi|190010910		(R)YPDLR
2	-----	00149	2	0.0	600.4	1.32	0.06	449	9	7/8	gi|190010910	+1	(K)RVAVR
916	-----	04462	3	0.0	1042.5	1.31	**0.29**	562	3	19/36	gi|190010910		(R)VEVAGHTDSK
759	-----	03607	2	0.0	1445.8	1.30	**0.24**	257	4	11/26	gi|190010910		(R)LTNDAPFVTLGLGK
889	-----	04324	2	0.0	1445.8	1.29	**0.18**	493	**1**	15/26	gi|190010910		(R)LTNDAPFVTLGLGK
832	-----	04028	2	0.0	1445.8	1.28	**0.16**	341	6	13/26	gi|190010910		(R)LTNDAPFVTLGLGK
376	-----	01929	2	0.0	1764.9	1.28	**0.27**	376	**1**	14/30	gi|190010910		(K)SEEEYNPISGGLADRK
653	-----	03157	2	0.0	941.4	1.26	**0.22**	476	13	10/14	gi|190010910		(K)GVNFDFDK
366	-----	01879	2	0.0	941.5	1.25	**0.36**	221	12	8/14	gi|190010910		(K)GVNFDFDK
379	-----	01952	2	0.0	941.4	1.23	**0.17**	261	14	9/14	gi|190010910		(K)GVNFDFDK
377	-----	01943	3	0.0	1042.5	1.22	**0.13**	**627**	**1**	19/36	gi|190010910		(R)VEVAGHTDSK
240	-----	01418	3	0.0	1042.5	1.15	**0.20**	**651**	14	16/36	gi|190010910		(R)VEVAGHTDSK
779	-----	03711	2	0.0	708.4	1.10	0.04	243	5	7/10	gi|190010910		(R)AEVAYR
786	-----	03763	2	0.0	1445.8	1.06	0.07	457	**1**	14/26	gi|190010910		(R)LTNDAPFVTLGLGK
340	-----	01767	3	0.0	1042.5	0.95	0.07	**609**	4	15/36	gi|190010910		(R)VEVAGHTDSK
93	-----	00811	4	0.0	1919.9	0.94	**0.10**	463	**1**	37/102	gi|190010910		(R)VEVAGHTDSKGTDAYNQK
792	-----	03789	2	0.0	708.4	0.93	**0.12**	379	**2**	8/10	gi|190010910		(R)AEVAYR
859	-----	04170	2	0.0	708.4	0.72	**0.17**	170	8	6/10	gi|190010910		(R)AEVAYR

Table S2. LC-MS/MS analysis of the band B protein spot (Fig. 1, 31-kDa)

>Smlt0955 (OmpA)

```
MNKKILTAAL LGGLAFAQAA SAQEFDDRWY LTGSAGFNFQ DSDRLTNDAP FVTLGLGKFI SPNWSLDGEL NYQNPNFDAN
KDMNWSQYGV SLDLRRHFIK EGRGWNPYLL AGLGYQKSEE EYNPISGGLA DRKDGNFAAK VGVGLQTTFE KRVAVRAEVA
YRADFDDQSV NPKRAGNDES WFGDVLASVG VVIPLGPAPV AAAPAPAPVA PSCADLDDDG DGVNNCDDKC PNSQPGQTIG
PDGCPVPVSI DLKGVNFDFD KSNLRPDAVA ILSEATEILK RYPDLRVEVA GHTDSKGTDA YNQKLSERRA TAVYNYLTKN
GVDAGRLVGP IGYGESRPIA PNTNPDGSDN PEGRAKNRRT ELNVQN
```

#	zBP	File	z	dM	MH+	Xcorr	dCn	Sp	RSp	Ions	Reference	( )Sequence
175	-----	02528	3	0.0	2683.2	6.62	0.57	4796	1	45/88	law54_04420	(K)FISPNWSLDGELNYQNPNFDANK
182	-----	02604	3	0.0	2683.2	6.10	0.55	3545	1	43/88	law54_04420	(K)FISPNWSLDGELNYQNPNFDANK
202	-----	02824	4	0.0	4364.0	5.74	0.70	2395	1	62/216	law54_04420	(K)FISPNWSLDGELNYQNPNFDANKDM*NWSQYGVSLDLR
78	-----	01894	2	0.0	1636.7	5.70	0.64	2213	1	24/28	law54_04420	(K)SEEEYNPISGGLADR
206	-----	02859	4	0.0	4364.0	5.70	0.74	1938	1	56/216	law54_04420	(K)FISPNWSLDGELNYQNPNFDANKDM*NWSQYGVSLDLR
197	-----	02786	4	0.0	4364.0	5.46	0.67	2564	1	59/216	law54_04420	(K)FISPNWSLDGELNYQNPNFDANKDM*NWSQYGVSLDLR
183	-----	02616	2	0.0	1579.8	5.12	0.45	2103	1	23/26	law54_04420	(R)GWNPYLLAGLGYQK
205	-----	02857	2	0.0	1863.8	5.04	0.75	1992	1	23/30	law54_04420	(R)WYLTGSAGFNFQDSDR
176	-----	02530	2	0.0	2683.2	5.03	0.74	926	1	21/44	law54_04420	(K)FISPNWSLDGELNYQNPNFDANK
204	-----	02846	3	0.0	4364.0	4.98	0.66	1636	1	43/144	law54_04420	(K)FISPNWSLDGELNYQNPNFDANKDM*NWSQYGVSLDLR
57	-----	01785	2	0.0	1764.8	4.93	0.69	1995	1	25/30	law54_04420	(K)SEEEYNPISGGLADRK
196	-----	02778	3	0.0	4364.0	4.87	0.59	894	1	38/144	law54_04420	(K)FISPNWSLDGELNYQNPNFDANKDM*NWSQYGVSLDLR
213	-----	02936	3	0.0	4364.0	4.86	0.67	1412	1	40/144	law54_04420	(K)FISPNWSLDGELNYQNPNFDANKDM*NWSQYGVSLDLR
104	-----	02060	2	0.0	1636.7	4.83	0.65	1589	1	22/28	law54_04420	(K)SEEEYNPISGGLADR
185	-----	02666	2	0.0	1863.8	4.82	0.70	2140	1	24/30	law54_04420	(R)WYLTGSAGFNFQDSDR
221	-----	03563	2	0.0	1863.8	4.69	0.76	1501	1	22/30	law54_04420	(R)WYLTGSAGFNFQDSDR
164	-----	02426	2	0.0	1699.8	4.67	0.65	2326	1	23/26	law54_04420	(K)DM*NWSQYGVSLDLR
86	-----	01970	2	0.0	1636.7	4.63	0.67	2001	1	24/28	law54_04420	(K)SEEEYNPISGGLADR
193	-----	02750	2	0.0	1863.8	4.62	0.72	2297	1	25/30	law54_04420	(R)WYLTGSAGFNFQDSDR
49	-----	01713	3	0.0	1924.9	4.53	0.63	2219	1	41/64	law54_04420	(R)AEVAYRADFDDQSVNPK
226	-----	04586	4	0.0	4520.1	4.51	0.51	1043	1	52/222	law54_04420	(K)FISPNWSLDGELNYQNPNFDANKDM*NWSQYGVSLDLRR
51	-----	01715	2	0.0	1764.8	4.44	0.70	1575	1	24/30	law54_04420	(K)SEEEYNPISGGLADRK
43	-----	01684	2	0.0	1924.9	4.31	0.62	1410	1	21/32	law54_04420	(R)AEVAYRADFDDQSVNPK
81	-----	01913	3	0.0	1636.7	4.22	0.59	1748	1	36/56	law54_04420	(K)SEEEYNPISGGLADR
179	-----	02590	2	0.0	1863.8	4.19	0.65	1532	1	21/30	law54_04420	(R)WYLTGSAGFNFQDSDR
97	-----	01999	4	0.0	2632.3	4.15	0.54	2190	1	55/138	law54_04420	(K)CPNSQPGQTIGPDGCPVPVSIDLK
189	-----	02688	5	0.0	4520.1	4.15	0.46	3397	1	76/296	law54_04420	(K)FISPNWSLDGELNYQNPNFDANKDM*NWSQYGVSLDLRR
123	-----	02198	2	0.0	1636.8	4.12	0.65	1546	1	21/28	law54_04420	(K)SEEEYNPISGGLADR
166	-----	02432	3	0.0	1699.8	4.05	0.40	1577	1	29/52	law54_04420	(K)DM*NWSQYGVSLDLR
209	-----	02880	2	0.0	2683.3	3.95	0.65	560	1	18/44	law54_04420	(K)FISPNWSLDGELNYQNPNFDANK
216	-----	02962	4	0.0	4520.1	3.94	0.45	1084	1	52/222	law54_04420	(K)FISPNWSLDGELNYQNPNFDANKDM*NWSQYGVSLDLRR
203	-----	02828	3	0.0	4348.0	3.90	0.66	687	1	36/144	law54_04420	(K)FISPNWSLDGELNYQNPNFDANKDMNWSQYGVSLDLR
188	-----	02682	3	0.0	4520.1	3.83	0.65	697	1	40/148	law54_04420	(K)FISPNWSLDGELNYQNPNFDANKDM*NWSQYGVSLDLRR
184	-----	02618	3	0.0	1579.8	3.82	0.47	1633	1	29/52	law54_04420	(R)GWNPYLLAGLGYQK
198	-----	02810	4	0.0	4520.1	3.80	0.56	1299	1	57/222	law54_04420	(K)FISPNWSLDGELNYQNPNFDANKDM*NWSQYGVSLDLRR
220	-----	03554	4	0.0	4520.1	3.78	0.57	1213	1	57/222	law54_04420	(K)FISPNWSLDGELNYQNPNFDANKDM*NWSQYGVSLDLRR
187	-----	02670	4	0.0	4520.1	3.78	0.52	1474	1	61/222	law54_04420	(K)FISPNWSLDGELNYQNPNFDANKDM*NWSQYGVSLDLRR
66	-----	01844	3	0.0	2468.2	3.74	0.58	1174	1	33/88	law54_04420	(K)SEEEYNPISGGLADRKDGNFAAK
155	-----	02376	2	0.0	1445.8	3.68	0.59	727	1	19/26	law54_04420	(R)LTNDAPFVTLGLGK
69	-----	01853	2	0.0	2468.2	3.65	0.65	792	1	21/44	law54_04420	(K)SEEEYNPISGGLADRKDGNFAAK
173	-----	02502	2	0.0	1445.8	3.59	0.46	781	1	19/26	law54_04420	(R)LTNDAPFVTLGLGK
215	-----	02961	4	0.0	4520.1	3.58	0.52	1018	1	53/222	law54_04420	(K)FISPNWSLDGELNYQNPNFDANKDM*NWSQYGVSLDLRR
191	-----	02737	4	0.0	4520.1	3.52	0.59	865	1	51/222	law54_04420	(K)FISPNWSLDGELNYQNPNFDANKDM*NWSQYGVSLDLRR
210	-----	02884	4	0.0	4520.1	3.51	0.56	1433	1	57/222	law54_04420	(K)FISPNWSLDGELNYQNPNFDANKDM*NWSQYGVSLDLRR
160	-----	02402	3	0.0	1445.8	3.48	0.62	1535	1	29/52	law54_04420	(R)LTNDAPFVTLGLGK
214	-----	02959	2	0.0	1178.6	3.44	0.47	1267	1	17/20	law54_04420	(K)VGVGLQTTFEK
99	-----	02017	2	0.0	1178.6	3.41	0.43	1357	1	17/20	law54_04420	(K)VGVGLQTTFEK
85	-----	01958	2	0.0	1411.7	3.38	0.50	1368	1	18/22	law54_04420	(K)GVNFDFDKSNLR
113	-----	02128	2	0.0	1178.6	3.31	0.49	1303	1	17/20	law54_04420	(K)VGVGLQTTFEK
55	-----	01766	2	0.0	1334.7	3.29	0.41	792	1	15/22	law54_04420	(K)VGVGLQTTFEKR
72	-----	01869	2	0.0	1178.6	3.25	0.51	1073	1	17/20	law54_04420	(K)VGVGLQTTFEK
222	-----	03564	2	0.0	1178.6	3.22	0.46	1470	1	17/20	law54_04420	(K)VGVGLQTTFEK
52	-----	01737	3	0.0	1334.7	3.21	0.49	1139	1	28/44	law54_04420	(K)VGVGLQTTFEKR
10	-----	01252	2	0.0	1235.6	3.17	0.60	1491	1	17/20	law54_04420	(R)ADFDDQSVNPK
24	-----	01514	2	0.0	1764.8	3.12	0.70	445	1	20/30	law54_04420	(K)SEEEYNPISGGLADRK
218	-----	03174	4	0.0	5697.7	3.09	0.21	993	1	60/330	law54_04420	(K)RAGNDESWFGDVLASVGVVIPLGPAPVAAAPAPAPVAPSCADLDDDGDGV NNCDDK
190	-----	02704	3	0.0	4504.1	3.07	0.46	499	1	36/148	law54_04420	(K)FISPNWSLDGELNYQNPNFDANKDMNWSQYGVSLDLRR
225	-----	04582	2	0.0	1445.8	3.07	0.50	775	1	19/26	law54_04420	(R)LTNDAPFVTLGLGK
37	-----	01648	2	0.0	1636.7	3.04	0.58	1052	1	19/28	law54_04420	(K)SEEEYNPISGGLADR
82	-----	01941	2	0.0	1178.6	3.01	0.50	1326	1	17/20	law54_04420	(K)VGVGLQTTFEK
96	-----	01998	3	0.0	2632.3	3.00	0.42	508	1	35/92	law54_04420	(K)CPNSQPGQTIGPDGCPVPVSIDLK
17	-----	01407	2	0.0	1133.6	2.95	0.40	1254	1	15/18	law54_04420	(R)VAVRAEVAYR
70	-----	01855	2	0.0	1334.7	2.95	0.40	691	1	14/22	law54_04420	(K)VGVGLQTTFEKR
83	-----	01948	2	0.0	1334.7	2.95	0.43	794	1	15/22	law54_04420	(K)VGVGLQTTFEKR
11	-----	01341	2	0.0	1235.6	2.92	0.46	1147	1	18/20	law54_04420	(R)ADFDDQSVNPK
224	-----	04580	2	0.0	1178.6	2.83	0.50	1023	1	17/20	law54_04420	(K)VGVGLQTTFEK
112	-----	02114	2	0.0	1178.6	2.83	0.56	762	1	15/20	law54_04420	(K)VGVGLQTTFEK
227	-----	04627	2	0.0	1334.7	2.75	0.42	862	1	14/22	law54_04420	(K)VGVGLQTTFEKR

18	-----	01411	3	0.0	1133.6	**2.70**	**0.48**	**819**	**1**	24/36	law54_04420	(R)VAVRAEVAYR
8	-----	01244	2	0.0	1391.7	**2.59**	**0.41**	534	**2**	15/22	law54_04420	(R)ADFDDQSVNPKR
142	-----	02300	3	0.0	1855.9	**2.58**	**0.52**	**678**	**1**	25/56	law54_04420	(K)DM*NWSQYGVSLDLRR
65	-----	01843	4	0.0	2468.2	**2.57**	**0.54**	**877**	**1**	43/132	law54_04420	(K)SEEEYNPISGGLADRKDGNFAAK
44	-----	01694	2	0.0	1334.7	**2.54**	**0.33**	**614**	**1**	13/22	law54_04420	(K)VGVGLQTTFEKR
161	-----	02406	2	0.0	1839.9	2.49	**0.36**	154	4	14/28	law54_04420	(K)DMNWSQYGVSLDLRR
181	-----	02602	4	0.0	4520.1	2.47	**0.11**	**871**	**1**	47/222	law54_04420	(K)FISPNWSLDGELNYQNPNFDANKDM*NWSQYGVSLDLRR
47	-----	01705	3	0.0	1764.8	2.40	**0.40**	531	**1**	30/60	law54_04420	(K)SEEEYNPISGGLADRK
1	-----	00160	2	0.0	850.4	2.22	**0.17**	512	**1**	12/14	law54_04420	(R)KDGNFAAK
201	-----	02820	4	0.0	4348.0	2.20	**0.30**	**1076**	**2**	49/216	law54_04420	(K)FISPNWSLDGELNYQNPNFDANKDMNWSQYGVSLDLR
3	-----	00506	2	0.0	722.3	2.16	**0.51**	**637**	**1**	11/12	law54_04420	(K)DGNFAAK
211	-----	02888	3	0.0	2683.3	2.15	**0.28**	**811**	**1**	28/88	law54_04420	(K)FISPNWSLDGELNYQNPNFDANK
56	-----	01776	3	0.0	1764.8	2.08	**0.48**	569	**1**	29/60	law54_04420	(K)SEEEYNPISGGLADRK
156	-----	02378	3	0.0	1855.9	2.04	**0.41**	**642**	**1**	24/56	law54_04420	(K)DM*NWSQYGVSLDLRR
127	-----	02227	3	0.0	1855.9	2.01	**0.32**	583	**1**	23/56	law54_04420	(K)DM*NWSQYGVSLDLRR
59	-----	01810	3	0.0	1334.7	2.00	**0.42**	**1083**	**1**	28/44	law54_04420	(K)VGVGLQTTFEKR
9	-----	01249	3	0.0	1391.7	1.94	**0.41**	**764**	**1**	27/44	law54_04420	(R)ADFDDQSVNPKR
84	-----	01951	3	0.0	1411.7	1.73	**0.24**	**657**	**1**	28/44	law54_04420	(K)GVNFDFDKSNLR
4	-----	00560	2	0.0	708.4	1.68	**0.27**	489	**2**	9/10	law54_04420	(R)AEVAYR
2	-----	00328	2	0.0	850.4	1.59	**0.27**	468	**1**	12/14	law54_04420	(R)KDGNFAAK
223	-----	03566	3	0.0	1334.7	1.39	**0.34**	**628**	**1**	25/44	law54_04420	(K)VGVGLQTTFEKR
128	-----	02238	2	0.0	1855.9	1.30	**0.32**	92	11	14/28	law54_04420	(K)DM*NWSQYGVSLDLRR
6	-----	00883	2	0.0	708.4	1.28	**0.39**	483	**1**	9/10	law54_04420	(R)AEVAYR

Table S3. LC-MS/MS analysis of the band C protein spot (Fig. 1, 28-kDa)

>Smlt4119

MRPVPTLLAL SLLAAGASFA NAAHAAEGDD RFAIR**LGAMN IDSDNTLRGS TTVAGQDISL NQDFKLGGKE WEPR**IDGMFR
ISNR**QRLLFN YFKYDK**DR**RE TLDQGISFGG ENVPAGSFVK GELK**YQVASL VYDYSVVDTD TFDLGLQIGG EYAKVSTK**GY**
ADLGTVYEGQ FLDEKADGVA PVVGARMTFT PSERWMITLQ GQYLNTRWGS FDDYKGDLSR ANAIVDYRFT K**NFGVFAGYD**
WFKLDVDKKG SDGTVGLKQE FKGPVAGISF VF

#	zBP	File	z	dM	MH+	Xcorr	dCn	Sp	RSp	Ions	Reference	( )Sequence
163	-----	02425	3	0.0	2897.4	**7.19**	**0.70**	**2531**	**1**	48/108	law54_20185	(K)GYADLGTVYEGQFLDEKADGVAPVVGAR
125	-----	02222	3	0.0	2308.2	**6.51**	**0.70**	**2491**	**1**	44/84	law54_20185	(R)RETLDQGISFGGENVPAGSFVK
148	-----	02345	2	0.0	2152.1	**6.01**	**0.77**	**1843**	**1**	29/40	law54_20185	(R)ETLDQGISFGGENVPAGSFVK
91	-----	01982	2	0.0	1780.9	**5.07**	**0.72**	**2429**	**1**	25/32	law54_20185	(R)GSTTVAGQDISLNQDFK
159	-----	02387	2	0.0	1904.9	**4.83**	**0.66**	**1813**	**1**	24/32	law54_20185	(K)GYADLGTVYEGQFLDEK
93	-----	01991	2	0.0	1545.7	**4.78**	**0.68**	**1609**	**1**	22/24	law54_20185	(R)WGSFDDYKGDLSR
147	-----	02342	2	0.0	1639.8	**4.15**	**0.70**	**2273**	**1**	21/24	law54_20185	(R)WM*ITLQGQYLNTR
149	-----	02346	3	0.0	2152.1	**4.03**	**0.58**	**1565**	**1**	39/80	law54_20185	(R)ETLDQGISFGGENVPAGSFVK
71	-----	01866	2	0.0	1419.7	**4.00**	**0.46**	**1139**	**1**	20/24	law54_20185	(R)LGAMNIDSDNTLR
207	-----	02872	2	0.0	1450.7	**3.47**	**0.65**	**1283**	**1**	19/22	law54_20185	(K)NFGVFAGYDWFK
95	-----	01993	3	0.0	1780.9	**3.47**	**0.52**	**1373**	**1**	35/64	law54_20185	(R)GSTTVAGQDISLNQDFK
146	-----	02336	2	0.0	1350.7	**3.46**	**0.54**	**1168**	**1**	16/18	law54_20185	(R)LLFNYFKYDK
153	-----	02364	3	0.0	2579.3	**3.45**	**0.64**	**927**	**1**	35/96	law54_20185	(R)ETLDQGISFGGENVPAGSFVKGELK
200	-----	02814	2	0.0	1525.8	**3.39**	**0.63**	**831**	**1**	19/26	law54_20185	(K)QEFKGPVAGISFVF
192	-----	02738	3	0.0	2149.1	**3.39**	**0.48**	**710**	**1**	32/68	law54_20185	(K)NFGVFAGYDWFKLDVDKK
30	-----	01580	2	0.0	1435.7	**3.22**	**0.43**	**1384**	**1**	20/24	law54_20185	(R)LGAM*NIDSDNTLR
195	-----	02771	2	0.0	2340.2	**2.93**	**0.66**	**1077**	**1**	22/44	law54_20185	(K)GSDGTVGLKQEFKGPVAGISFVF
212	-----	02892	3	0.0	2021.0	**2.85**	**0.43**	**702**	**1**	28/64	law54_20185	(K)NFGVFAGYDWFKLDVDK
12	-----	01356	2	0.0	1011.6	**2.84**	**0.56**	**885**	**1**	17/20	law54_20185	(K)ADGVAPVVGAR
15	-----	01404	2	0.0	1071.6	**2.67**	**0.42**	**689**	**1**	13/16	law54_20185	(K)LGGKEWEPR
79	-----	01901	2	0.0	1017.4	**2.59**	**0.51**	512	**1**	13/14	law54_20185	(R)WGSFDDYK
136	-----	02265	3	0.0	1228.7	**2.56**	**0.52**	**809**	**1**	22/32	law54_20185	(R)QRLLFNYFK
194	-----	02761	3	0.0	2340.2	**2.50**	**0.67**	488	**1**	29/88	law54_20185	(K)GSDGTVGLKQEFKGPVAGISFVF
73	-----	01874	2	0.0	1435.7	2.48	**0.30**	**827**	**1**	16/24	law54_20185	(R)LGAM*NIDSDNTLR
92	-----	01985	3	0.0	1545.7	2.48	**0.39**	**1504**	**1**	29/48	law54_20185	(R)WGSFDDYKGDLSR
16	-----	01405	3	0.0	1493.8	2.43	**0.37**	**1417**	**1**	32/52	law54_20185	(K)KGSDGTVGLKQEFK
27	-----	01529	2	0.0	1365.7	2.41	**0.44**	**962**	**1**	18/24	law54_20185	(K)GSDGTVGLKQEFK
21	-----	01474	2	0.0	921.5	2.35	**0.50**	**910**	**1**	13/14	law54_20185	(R)ANAIVDYR
169	-----	02462	2	0.0	944.5	2.07	**0.43**	368	**1**	11/12	law54_20185	(R)LLFNYFK
28	-----	01530	3	0.0	1365.7	1.80	**0.41**	**623**	**1**	27/48	law54_20185	(K)GSDGTVGLKQEFK
7	-----	01174	2	0.0	984.4	1.64	**0.54**	243	**1**	10/14	law54_20185	(R)M*TFTPSER
14	-----	01398	3	0.0	1071.6	1.59	**0.20**	525	6	20/32	law54_20185	(K)LGGKEWEPR

Table S4 Antibiotic susceptibilities of *S. maltophilia* KJ and KJΔOmpA$_{299\text{-}356}$

	MIC (μg/ml)			
	Quinolone			SXT
	CIP	LEV	MOX	
KJ	0.19	0.125	0.064	0.19
KJΔOmpA$_{299\text{-}356}$	0.25	0.125	0.064	0.19

[a]MIC, Minimal Inhibitory Concentration

Abbreviation: CIP, ciprofloxacin; LEV, levofloxacin; MOX, moxifloxacin; SXT, trimethoprim-sulfamethoxazole.

Table S6 Transcriptome analysis of the PG homeostasis-associated genes in wild-type KJ and *ompA* mutant, KJΔOmpA$_{299\text{-}356}$

Locus	Protein	TPM		Fold change
		KJ	KJΔOmpA$_{299\text{-}356}$	
**(A) Genes involved in PG homeostasis**				
PG biosynthesis pathway				
Smlt0753	MraY	158.53	166.02	+1.05
Smlt0755	MurG	308.35	511.52	+1.66
Smlt3826	PBP1a	73.09	57.42	-1.27
Smlt3681	PBP1b	176.83	207.80	+1.18
Smlt3602	PBP1c	23.91	11.69	-2.05
Smlt4056	PBP2	33.40	55.32	+1.66
Smlt0750	PBP3	304.86	340.82	+1.12
Smlt0462	PBP4	13.01	20.45	+1.57
Smlt4050	PBP6	401.70	537.31	+1.34
PG metabolism pathway				
Smlt0154	Amidase	33.86	15.16	-2.23
Smlt0155	MltA	65.32	87.81	+1.34
Smlt4052	MltB1	441.60	470.88	+1.07
Smlt4650	MltB2	55.00	95.37	+1.73
Smlt0994	MltD1	193.65	264.08	+1.36
Smlt3434	MltD2	191.35	178.71	-1.07
Smlt4007	Slt	85.09	238.25	+2.80
Smlt0412	AmpN	131.49	125.02	-1.05
Smlt0413	AmpG	76.31	73.84	-1.03
Smlt1245	Opp	83.83	46.97	-1.78
PG recycling pathway				
Smlt3538	NagZ	147.78	100.14	-1.48
Smlt1562	AmpDI	104.47	106.88	+1.02
Smlt0415	AnmK	60.05	47.76	-1.26
Smlt0584	MupP	93.59	158.50	+1.69
Smlt1131	AmgK	77.82	75.35	-1.03
Smlt1130	MurU	41.33	58.09	+1.41
Smlt4020	NagA	31.80	147.72	**+4.65**
Smlt3414	GlmM	240.72	164.73	-1.46
Smlt4099	GlmS	352.12	537.00	+1.53

Smlt4108	GlmU	194.59	161.18	-1.21
Smlt3885	Mpl	175.01	275.47	+1.57
Smlt1119	MurA	349.25	290.93	-1.20
Smlt2127	MurB	53.21	50.96	-1.04
Smlt0756	MurC	452.60	627.95	+1.39
Smlt1170	MurD	56.71	129.44	+2.28
Smlt0751	MurE	293.25	391.23	+1.33
Smlt0752	MurF	130.78	185.13	+1.42
**(B) β-lactamase genes**				
Smlt2667	L1	12.64	23.18	+1.83
Smlt3722	L2	19.69	75.12	**+3.82**

[a]TPM, Transcripts Per Kilobase Million

[b]Negative fold changes represent genes that were significantly downregulated in KJΔOmpA$_{299-356}$, whereas positive fold changes represent upregulation in KJΔOmpA$_{299-356}$.

Table S7 Bacterial strains, plasmids, and primers used in this study

Strain, plasmid, or primer	Genotype or properties	Reference
S. maltophilia		
KJ	A clinical *S. maltophilia* isolate	1
KJ2	*S. maltophilia* KJ mutant of *L1 and L2* genes; *ΔL1, ΔL2*	2
KJΔOmpA$_{299-356}$	*S. maltophilia* KJ mutant of *ompA* gene; residues 299-356 deleted, named as KJΔOmpA previously	3
KJ2ΔOmpA$_{299-356}$	*S. maltophilia* KJ2 mutant of *ompA* gene; residues 299-356 deleted,	This study
KJL2-OmpAΔOmpA$_{299-356}$	*S. maltophilia* KJΔOmpA$_{299-356}$ with an inserted *ompA* gene downstream L2 gene	This study
KJΔNagAΔOmpA$_{299-356}$	*S. maltophilia* KJ mutant of *nagA* and *ompA* genes; *ΔnagA*, *ΔompA*	This study
KJΔNagA	*S. maltophilia* KJ mutant of *nagA* gene; *ΔnagA*	This study
KJΔRpoEΔOmpA$_{299-356}$	*S. maltophilia* KJ mutant of *rpoE* and *ompA* genes; *ΔrpoE*, *ΔompA*	This study
KJΔRpoPΔOmpA$_{299-356}$	*S. maltophilia* KJ mutant of *rpoP* and *ompA* genes; *ΔrpoP*, *ΔompA*	This study
KJΔRpoP	*S. maltophilia* KJ mutant of *rpoP* gene; *ΔrpoP*	This study
KJL2-RpoP	*S. maltophilia* KJ with an inserted *rpoP* gene downstream L2 gene	This study
E. coli		
DH5α	F- φ80d/*acZΔM15* Δ(*lacZYA-argF*)*U169 deoR recA1 endA1 hsdR17* (r_k^- m_k^+) *phoA supE44λ thi-1 gyrA96 relA1*	Invitrogen
S17-1	λ*pir*$^+$ mating strain	4
**Plasmids**		
pEX18Tc	*sacB oriT*, Tc^r	5

pΔNagA	pEX18Tc with an internal-deleted *nagA* gene; Tc^r	This study
pΔRpoE	pEX18Tc with an internal-deleted *rpoE* gene; Tc^r	This study
pΔRpoP	pEX18Tc with an internal-deleted *rpoP* gene; Tc^r	This study
pEXHH1	pEX18Tc with C-terminus of *L2* gene and downstream of *L2* gene; Tc^r	This study
pEXHH1-OmpA	pEXHH with an intact *ompA* gene; Tc^r	This study
pEXHH1-RpoP	pEXHH with an intact *rpoP* gene; Tc^r	This study
**Primers**		
NagA-F	GCA<u>AAGCTT</u>CTACCGCGCTATCAATGC	This study
NagA-R	CAG<u>GAATTC</u>GGTGTATCCCACCAG	This study
RpoP-F	CGG<u>AAGCTT</u>ATCCTTCAGGGACAA	This study
RpoP-R	AGC<u>GAATTC</u>GTGCGCTTGAGCTG	This study
RpoEN-F	GG<u>GAATT</u>CAGGGAGAGGACCACCA	This study
RpoEN-R	TTG<u>GGTACC</u>AGGACATCGAACGC	This study
RpoEC-F	GC<u>GGTACC</u>GAACAGTTCGACAGT	This study
RpoEC-R	GC<u>TCTAGA</u>TGGTTCTGCGATTCGT	This study
HH1N-F	CAC<u>AAGCTT</u>CACCAGCGACAACAC	This study
HH1N-R	TGA<u>GCATGC</u>TTACCTCATCCGATCAA	This study
HH1C-F	GCC<u>GAGCTC</u>TTGATCGGATGAGGTAA	This study
HH1C-R	CGG<u>GAATTC</u>CTTGCTGGTCAC	This study
HHOmpA-F	CT<u>TCTAGA</u>ACAAGAAGATCCT	This study
HHOmpA-R	GT<u>GAGCTC</u>GTGTCCTACTGGCA	This study
HHRpoP-F	CA<u>TCTAGA</u>CGGAGACCGTGTAATG	This study
HHRpoP-R	CC<u>GAGCTC</u>TTGACCAGCA	This study
NagAQ103-F	GTGATGACGTGGCAGTGATG	This study
NagAQ103-R	ATGTACGGGCCTTCCAGGT	This study
16S rRNA	GACCTTGCGCGATTGAATG	2
16S rRNA	CGGATCGTCGCCTTGGT	2

1. Hu RM, Huang KJ, Wu LT, Hsiao YJ, Yang TC. 2008. Induction of L1 and L2 beta-lactamases of *Stenotrophomonas maltophilia*. Antimicrob Agents Chemother 52:1198-1200.
2. Chen CH, Huang CC, Chung TC, Hu RM, Huang YW, Yang TC. 2011. Contribution of resistance nodulation-division efflux pump operon *smeU1-V-W-U2-X* to multidrug resistance of *Stenotrophomonas maltophilia*. Antimicrob Agents Chemother 55: 5826-5833.
3. Liao CH, Chang CL, Huang HH, Lin YT, Li LH, Yang TC. 2021. Interplay between OmpA and RpoN regulates flagellar synthesis in *Stenotrophomonas maltophilia*. Microorganisms 9:1216.
4. Simon R, O'Connell M, Labes M, Puhler A. 1986. Plasmid vector for the genetic analysis and manipulation of *Rhizobia* and other Gram-negative bacteria. Methods Enzymol 118:640-659.
5. Hoang TT, Karkhoff-Schweizer RR, Kutchma AJ, Schweizer HP. A broad-host-range Flp-FRT recombination system for site-specific excision of chromosomally-located DNA sequences: application for isolation of unmarked *Pseudomonas aeruginosa* mutants. 1998. Gene. 212:77-86.
